# Transcriptional regulation of anthocyanin biosynthesis in a high-anthocyanin resynthesized *Brassica napus* cultivar

**DOI:** 10.1186/s40709-018-0090-6

**Published:** 2018-11-26

**Authors:** Gayatri Goswami, Ujjal Kumar Nath, Jong-In Park, Mohammad Rashed Hossain, Manosh Kumar Biswas, Hoy-Taek Kim, Hye Ran Kim, Ill-Sup Nou

**Affiliations:** 1 Department of Horticulture, Sunchon National University, 255 Jungang-ro, Suncheon, Jeonnam 57922 South Korea; 2 Department of Genetics and Plant Breeding, Bangladesh Agricultural University, Mymensingh, Bangladesh; 3 University-Industry Cooperation Foundation, Sunchon National University, 255 Jungang-ro, Suncheon, Jeonnam 57922 South Korea; 4 Plant Systems Engineering Research Center, Korea Research Institute of Bioscience and Biotechnology, Daejeon, South Korea

**Keywords:** *Brassica napus*, Resynthesized, Microsynteny, Transgressive expression, Additive expression, Anthocyanins

## Abstract

**Background:**

Anthocyanins are plant secondary metabolites with key roles in attracting insect pollinators and protecting against biotic and abiotic stresses. They have potential health-promoting effects as part of the human diet. Anthocyanin biosynthesis has been elucidated in many species, enabling the development of anthocyanin-enriched fruits, vegetables, and grains; however, few studies have investigated *Brassica napus* anthocyanin biosynthesis.

**Results:**

We developed a high-anthocyanin resynthesized *B. napus* line, Rs035, by crossing anthocyanin-rich *B. rapa* (A genome) and *B. oleracea* (C genome) lines, followed by chromosome doubling. We identified and characterized 73 and 58 anthocyanin biosynthesis genes in silico in the A and C genomes, respectively; these genes showed syntenic relationships with 41 genes in *Arabidopsis thaliana* and *B. napus*. Among the syntenic genes, twelve biosynthetic and six regulatory genes showed transgressively higher expression in Rs035, and eight structural genes and one regulatory gene showed additive expression. We identified three early-, four late-biosynthesis pathways, three transcriptional regulator genes, and one transporter as putative candidates enhancing anthocyanin accumulation in Rs035. Principal component analysis and Pearson’s correlation coefficients corroborated the contribution of these genes to anthocyanin accumulation.

**Conclusions:**

Our study lays the foundation for producing high-anthocyanin *B. napus* cultivars. The resynthesized lines and the differentially expressed genes we have identified could be used to transfer the anthocyanin traits to other commercial rapeseed lines using molecular and conventional breeding.

**Electronic supplementary material:**

The online version of this article (10.1186/s40709-018-0090-6) contains supplementary material, which is available to authorized users.

## Background

Anthocyanins belong to the flavonoid class of secondary metabolites. These water-soluble pigments are widely distributed in plants, accumulating in the leaves, petals, sepals, and fruits to yield purple, red, and blue coloration [1, 2]. Anthocyanins attract insects for pollination and protect plants against biotic and abiotic stresses [3–7]. Eating foods enriched with anthocyanins might reduce inflammation and protect against certain types of cancer, cardiovascular, neurodegenerative, and various age-related diseases [8–10]. The health benefits of anthocyanins are believed to be closely linked to their antioxidant activities, through which they reduce the abundance of reactive oxygen species [11]. Anthocyanins also increase blood serum antioxidant levels and protect red blood cells from oxidative damage [8, 12].

*Brassica napus* is a natural allopolyploid with the genomic composition 2n = 4x = 38 (AACC). In addition to its international use as an oilseed and fodder crop, it is becoming increasingly popular as a vegetable, and a number of *B. napus* vegetable varieties have been developed [13]. For example, the leaves and edible flower buds and young stems of *B. napus* are consumed as vegetables in China and other Asian countries, and one subspecies is also commonly used as a root vegetable in Europe, the U.S., Canada, and Australia (rutabaga, *B. napus* subsp. *rapifera*). Considering the diverse health-promoting benefits of anthocyanins, *B. napus* cultivars with high anthocyanin contents may be popular with consumers; however, only one study has documented *B. napus* mutants with red or purple leaves [14]. In contrast, several high-anthocyanin varieties are available for two of the most popular leafy Brassicaceae vegetables, Chinese cabbage (*B. rapa* ssp. *pekinensis*) and cabbage (*B. oleracea* var. *capitata*).

**Fig. 1:**
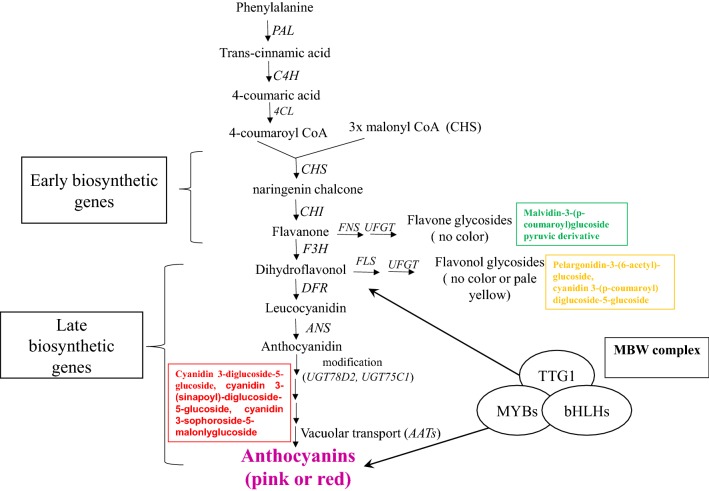
Schematic and simplified diagram of the anthocyanin biosynthesis pathway leading to the accumulation of anthocyanin in *Brassica*. The pathway is divided into three major parts; the upstream phenylpropanoid pathway, the early biosynthetic pathway, and the late biosynthetic pathway, based on the types of genes involved in each stage. The possible activation sites of the regulatory MBW complexes are indicated. Details of the genes involved in each stage are provided in Table 2. *PAL* phenylalanine ammonia lyase, *C4H* cinnamate-4-hydroxylase, *4CL* 4-coumaratel-CoA ligase, *CHS* chalcone synthase, *CHI* chalcone isomerase, *F3H* flavanone-3-hydroxylase, *FLS* flavonol synthase, *DFR* dihydroflavanol reductase, *ANS* anthocyanidin synthase, *UGT* uradin glycosyltransferase, *MYB* myeloblastosis family of transcription factors, *bHLH* basic helix-loop-helix

Understanding the regulation of anthocyanin biosynthesis is vital for enabling us to develop anthocyanin-enriched fruits and vegetables. Anthocyanin biosynthesis begins in the cytoplasm under the coordinated regulation of the structural genes, which encode several enzymes and regulatory transcription factors [15]. Glutathione *S*-transferases are involved in the export of the anthocyanins from the cytoplasm to the vacuole, where they are permanently stored [16, 17]. The anthocyanin biosynthesis pathway can be divided into three major consecutive phases (Fig. 1). In the first phase, phenylalanine is converted into trans-cinnamic acid and 4-coumaroyl-CoA by phenylalanine ammonia-lyase (PAL), cinnamate 4-hydroxylase (C4H), and 4-coumarate-CoA ligase (4CL), respectively [18–20]. Next, a series of enzymatic reactions catalyzed by chalcone synthase (CHS), chalcone isomerase (CHI), flavanone-3-hydroxylase (F3H) lead to the formation of dihydroflavonol from one molecule of coumarate-CoA and three molecules of malonyl-CoA. In the third stage, many types of anthocyanidins are formed from the dihydroflavonols, in reactions catalyzed by dihydroflavonol 4-reductase (DFR) and anthocyanidin synthase (ANS). Anthocyanidins are finally modified by a series of glycosylation and methylation steps to form anthocyanins, catalyzed by UDP-glucose:flavonoid glucosyltransferase (UFGT) and methyl transferase (MT) [21, 22].

Both structural and regulatory genes are involved in anthocyanin accumulation in *Arabidopsis thaliana* [19, 23, 24]. Primarily, anthocyanin biosynthesis is regulated at the transcriptional level; previous studies demonstrated that the expression patterns of the anthocyanin structural genes are mainly determined by the R2R3-MYB (myeloblastosis family of transcription factors), basic helix-loop-helix (bHLH), and WD40-repeat transcription factors, and by their coordinated interactions [25]. Lang et al. [26] reported that R2R3-MYB and bHLH transcription factors can bind to specific *cis*-acting elements in promoter regions of the structural genes and regulate their expression. The MBW (R2R3-MYB, bHLH, and WD40) complex is unique to plants, but this transcriptional regulatory system may vary between monocot and dicot plants; all three members of the MBW complex are not required for anthocyanin biosynthesis in every species [21, 22].

Recent studies have revealed that, in addition to the regulatory genes, the structural genes could also play a vital role in the accumulation of high levels of anthocyanin in several plant species [18, 27]. Zhang et al. [27] reported that most of the structural genes, including *PAL, CHS*, *F3H*, *F3′H*, *DFR*, *ANS*, and *anthocyanin 5*-*0*-*glucosyltransferase* (*5GT*) were significantly upregulated in purple kohlrabi (*Brassica oleracea* var. *gongylodes*), while only two regulatory genes, *transparent testa8* (*TT8*) and *production of anthocyanin pigment2* (*PAP2*) were upregulated. Previous reports showed that most of the anthocyanin pathway structural genes are upregulated in *B. rapa* varieties with high anthocyanin levels [28, 29]. The increased expression of structural genes in red cabbage was found to be coordinated by a bHLH, *TT8*, and a MYB transcription factor gene, *MYB2* [11].

Allopolyploidization, the merging of two or more divergent genomes into a common background, enhances the genomic diversity of the resulting organism [30–32]. Allopolyploids arise from interspecific or intergenic hybridization followed by chromosome doubling, and can act as a bridge species for gene introgression into important crop species. The genus *Brassica* contains a number of diploid and allopolyploid species. Among them, six agriculturally important species can be categorized into three basic diploid genomes (A, B, and C; n = 10, 8, and 9, respectively) and their allopolyploid hybrids (AB, AC, and BC), as confirmed using cytogenetics [33]. *Brassica napus* (AACC; 2n = 38) is an allopolyploid that evolved following the hybridization of its ancestors *B. rapa* (AA; 2n = 20) and *B. oleracea* (CC, 2n = 18) [33]. Sequence variations within chromosome segments showed that some of the A- and C-genome components of *B. napus* have undergone genetic changes since this hybridization event [34–36]. In contrast, resynthesized *B. napus* (produced by crossing *B. rapa* and *B. oleracea* followed by embryo rescue and chromosome doubling) was shown to undergo extensive chromosomal rearrangements immediately after allopolyploidization [37–39]. In the present study, we therefore found a wide variation in anthocyanin contents in resynthesized *B. napus* lines rather than either of the parents or intermediate of the parental lines.

Here, we characterized the structural and regulatory genes of anthocyanin biosynthesis in a purple-colored resynthesized *B. napus* line. We developed an anthocyanin-enriched allopolyploid *B. napus* by crossing a red-green Chinese cabbage (*B. rapa* ssp. *pekinensis*, AA genome) and a red cabbage (*B. oleracea* var. *capitata*, DD genome), followed by embryo rescue and chromosome doubling using colchicine. The aims of this study were to (1) select a stable anthocyanin-enriched resynthesized *B. napus* line developed by interspecies hybridization, (2) investigate the impact of the total anthocyanin content on the purple pigmentation of the leaves, (3) predict the candidate genes responsible for the purple leaf trait in *B. napus* using expression profiling and determine the expression patterns of those genes, and (4) determine the relationship between the different gene expression profiles and total and constituent anthocyanin contents in *B. napus*.

## Results

### Selection of an anthocyanin-enriched resynthesized *B. napus* line

**Fig. 2:**
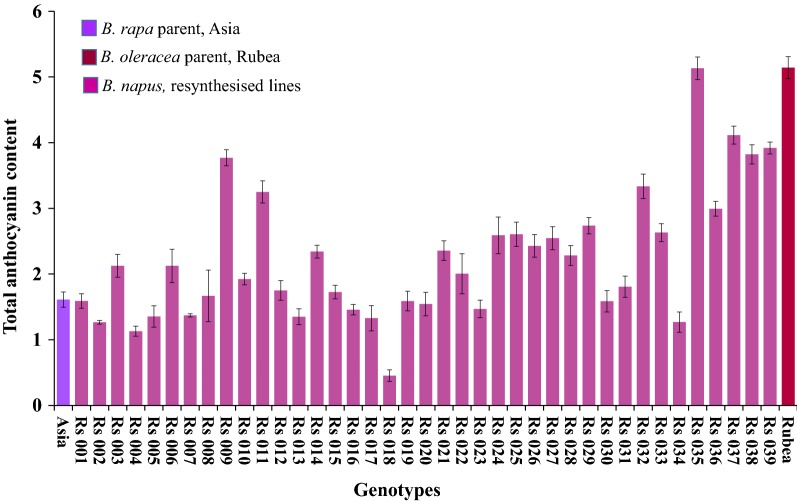
Total anthocyanin contents in 39 resynthesized *B. napus* lines along with their parental lines

The anthocyanin-enriched *B. napus* line was selected based on visual observations and spectrophotometric of the 39 resynthesized (Rs) lines developed by crossing a red-green Chinese cabbage inbred line (Asia) and a red cabbage inbred line (Rubea) followed by embryo rescue and chromosome doubling. Allopolyploids resulting from the interspecies cross were confirmed using the COS1078 marker and by checking their DNA contents using flow cytometry (data not shown). The Rs lines had a wide variety of total anthocyanin contents (< 0.5 to > 4.5 mg g^−1^ FW) (Fig. 2). Five Rs lines with total anthocyanin contents > 4.0 mg g^−1^ FW were selected and evaluated in a five-replicate trial during the next season. Of these, Rs035 had the highest total anthocyanin content (5.13 ± 0.17 mg g^−1^ FW) and was selected for further expression profiling using qRT-PCR.

### Evaluation of the anthocyanin profiles in *Brassica* lines

The total anthocyanin contents of contrastingly pigmented *Brassica* lines (Asia, Rubea, the red Rs line Rs035, and the green Rs line Rs306) were measured. The total anthocyanin contents were higher in the three red lines (Asia, Rs035, and Rubea; Additional file 5: Figure S1). The Rs035 line had 2.6-fold higher anthocyanin content compared to its *B. rapa* parent (Asia). Moreover, the same line had comparatively lower anthocynin than its *B. oleracea* parent (cv. Rubea) (Fig. 2). No anthocyanin was detected in the resynthesized green *B. napus* line Rs306.

We carried out HPLC to investigate the anthocyanin composition of our contrasting *Brassica* plants. A total of six anthocyanin components were detected in the four lines (Table 1), of which cyanidin-3-diglucoside-5-glucoside, cyanidin-3-(sinapoyl)-diglucoside-5-glucoside, cyanidin-3-sophoroside-5-malonylglucoside, malvidin-3-(*p*-coumaroyl)glucoside pyruvic derivative, and pelargonidin-3-(6-acetyl)-glucoside were the major anthocyanin components in Rs035. Four anthocyanin components were detected in the Chinese cabbage line Asia: cyanidin-3-diglucoside-5-glucoside, cyanidin-3-(sinapoyl)-diglucoside-5-glucoside, cyanidin-3-sophoroside-5-malonylglucoside, and pelargonidin-3-(6-acetyl)-glucoside. Among the six anthocyanin components, only three components, cyanidin-3-diglucoside-5-glucoside, cyanidin-3-(sinapoyl)-diglucoside-5-glucoside, and cyanidin-3-(*p*-coumaroyl)-diglucoside-5-glucoside, were found in the red cabbage line Rubea. None of the anthocyanin components were detected in the green Rs306 line (Table 1).

**Table 1:** Anthocyanin composition in leaves of *B. rapa* reddish line Asia (A-genome), resynthesis green *B. napus* line Rs306, resynthesis red *B. napus* line Rs035 and *B. oleracea* red cabbage line Rubea (C-genome) as detected by HPLC

Anthocyanin component	Anthocyanin levels (mg g^−1^ dry weight)			
	Asia	Rs306	Rs035	Rubea
Cyanidin3-diglucoside-5-glucoside	0.041	ND	0.235	0.59
Cyanidin3-(sinapoyl)-diglucoside-5-glucoside	0.105	ND	1.630	0.32
Cyanidin3-sophoroside-5-malonylglucoside	0.032	ND	0.070	ND
Malvidin3-(*p*-coumaroyl)glucoside pyruvic derivative	ND	ND	0.067	ND
Pelargonidin3-(6-acetyl)-glucoside	0.262	ND	0.256	ND
Cyanidin3-(*p*-coumaroyl) diglucoside-5-glucoside	ND	ND	ND	0.20

*ND* not detected

### Selection and in silico characterization of anthocyanin biosynthesis pathway genes

To predict and characterize the putative anthocyanin biosynthesis candidate genes in resynthesized *B. napus*, the anthocyanin pathway genes in *A. thaliana* were used to perform a syntenic gene search of the A (*B. rapa*) and C (*B. oleracea*) genomes from the BRAD database. The Bolbase and EnsemblPlants databases were also searched for anthocyanin genes in these species. A complementary method, Hidden Markov Model profiling, was performed to increase the accuracy of the identified genes within the *B. rapa* and *B. oleracea* genomes. The chromosomal position and the sub-genome status of these putative anthocyanin biosynthesis pathway genes, as well as the isoelectric points (Pi), molecular weights, and residual protein size of their predicted proteins, are presented in Additional file 1: Tables S1a and Additional file 2: Table S1b for *B. rapa* and *B. oleracea*, respectively.

We identified 73 putative anthocyanin pathway genes in *B. rapa* (A genome), of which 72 could be mapped onto one of its 10 chromosomes; chromosomes A01–A10 contained 3, 12, 12, 7, 10, 4, 6, 1, 12, and 5 putative genes, respectively. The gene, Bra035004, an ortholog of UGT79B1, is located on Scaffold000100 and could not be assigned to a chromosome. Of the other 72 putative genes, 21 were structural biosynthetic genes in the phenylpropanoid pathway, 18 were involved in early biosynthesis, six were late biosynthesis genes, 12 were positive regulators, 13 were negative regulators, and two encoded transporters (Additional file 1: Table S1a and Additional file 5: Figure S2a).

In *B. oleracea* (C genome), a total of 58 anthocyanin biosynthesis pathway genes were identified, of which 37 could be assigned to one of the nine chromosomes; chromosomes C01–C09 contained 2, 6, 4, 4, 1, 3, 6, 5, and 6 of the putative genes, respectively (Additional file 1: Table S1b and Additional file 5: Figure S2b). The other 21 genes were present on scaffolds, non-contiguous genomic sequences. The putative *B. oleracea* genes were categorized as 13 structural biosynthetic genes in the phenylpropanoid pathway, 13 early-biosynthesis genes, six late-biosynthesis genes, 24 positive and negative regulators, and two transporters (Additional file 2: Table S1b and Additional file 5: Figure S2b).

**Fig. 3:**
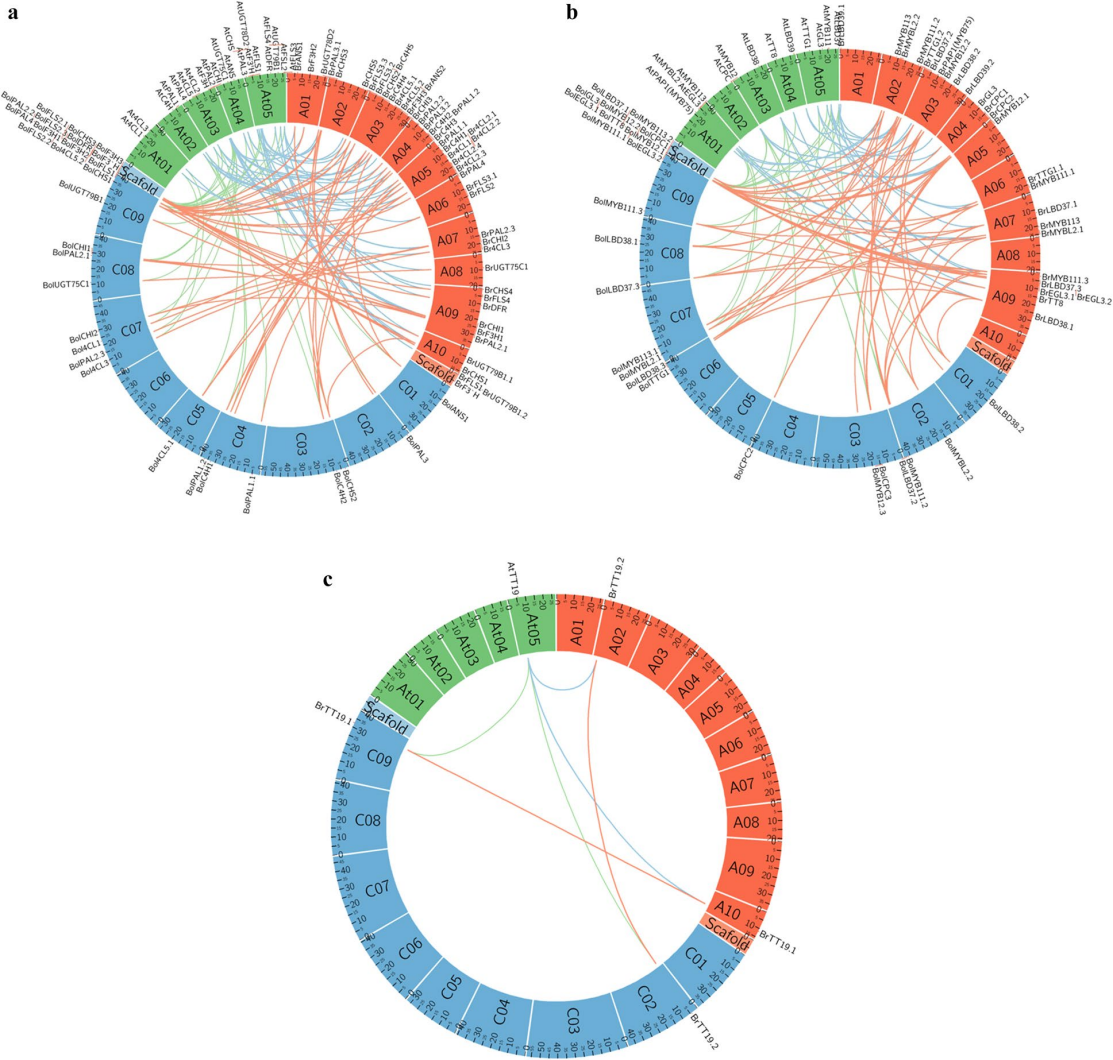
Microsyntenic analyses of anthocyanin biosynthesis pathway genes. **a** Structural, early-biosynthesis, and late-biosynthesis genes, **b** positive- and negative-regulatory genes, and **c** transporter genes in *Arabidopsis thaliana* (At, green), *Brassica rapa* (A, orange), and *B. oleracea* (C, blue)

Three separate microsynteny maps were constructed using *B. rapa*, *B. oleracea*, and *A. thaliana* orthologous pairs of structural, regulatory, and transporter genes in the anthocyanin pathway to investigate their evolutionary cues and genetic relationships among the three species (Fig. 3a–c). Based on our analysis, 41 structural genes in *B. rapa* were found to correspond to 24 *A. thaliana* orthologous genes, while 32 orthologous gene pairs were recognized between *B. rapa* and *B. oleracea* (Fig. 3a). Of the regulatory genes, the 24 identified in *B. rapa* showed syntenic relationships with 16 orthologous genes in *A. thaliana*, while 24 pairs of syntenic regulatory genes were found between *B. rapa* and *B. oleracea* (Fig. 3b). Meanwhile, two syntenic transporter genes corresponding to an *A. thaliana* ortholog were identified in both *B. rapa* and *B. oleracea* (Fig. 3c). These results suggest that the structural, regulatory, and transporter genes of the anthocyanin pathway maintain close syntenic relationships in *B. rapa*, *B. oleracea,* and *A. thaliana*.

### Prediction of putative candidate genes for the enhanced accumulation of anthocyanin

#### Expression profiling of anthocyanin biosynthesis genes

We carried out expression profiling of the anthocyanin biosynthesis genes to investigate their role in anthocyanin accumulation in the leaves of the contrasting *Brassica* lines. No striking effects were observed for the expression of the A-genome (*B. rapa*) and C-genome (*B. oleracea*) upstream (*PAL1*, *PAL2*, *C4H*, *4CL1*) and downstream (*UGT78D2*) genes of the anthocyanin pathway in the four lines examined (Additional file 5: Figure S3a), despite their differences in anthocyanin accumulation. However, the downstream gene *UGT78D2* was upregulated in the A genome of Rs035, with a seven-fold higher expression than in the green line Rs306. This result is expected because of *UGT78D2* gene has a preference of Cyanidin3-(sinapoyl)-diglucoside-5-glucoside metabolism and found higher amount in Rs035 compared to Rubea (Table 1).

**Fig. 4:**
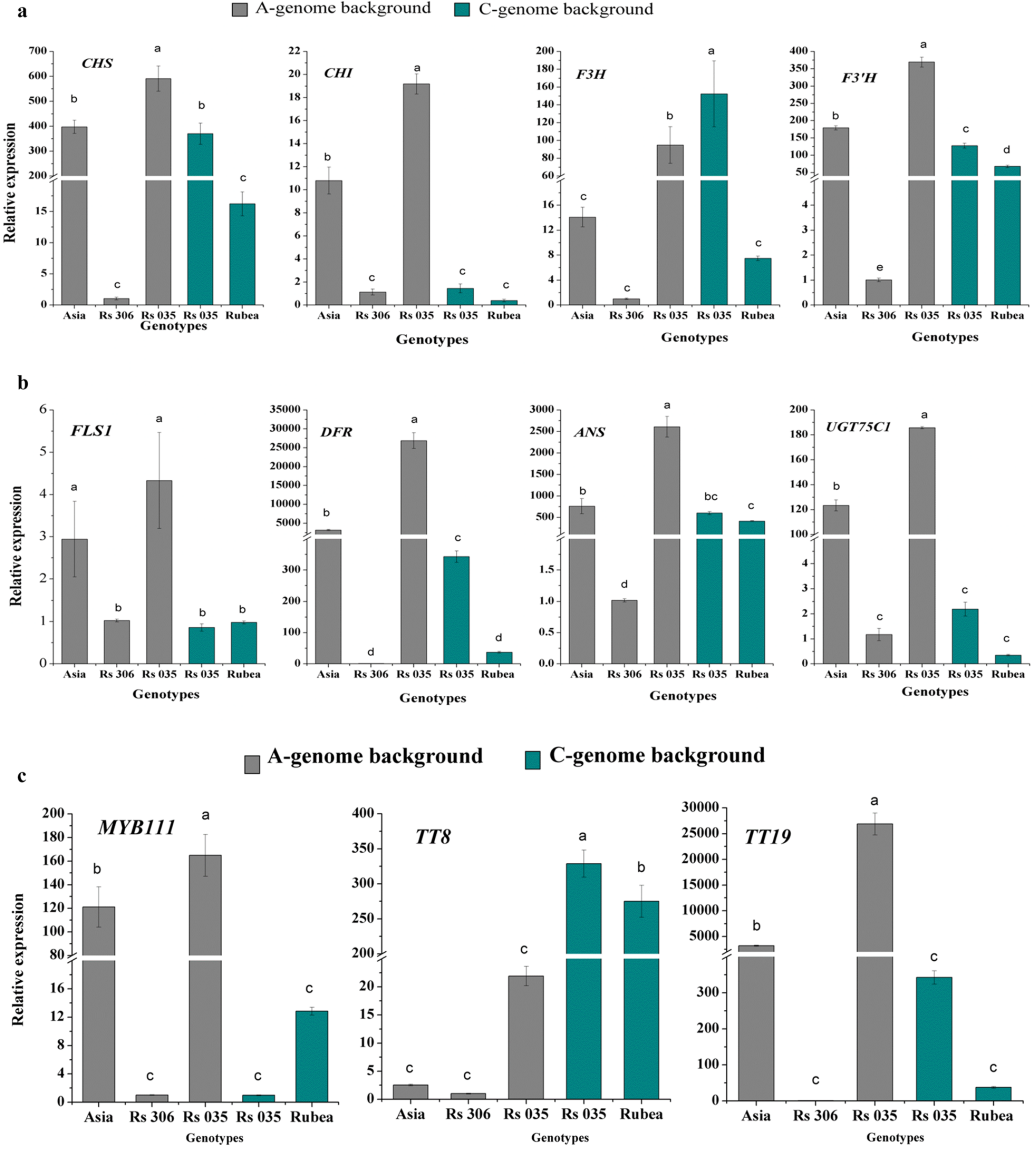
Expression patterns of the anthocyanin pathway genes. **a** Upstream structural and biosynthesis genes, **b** downstream biosynthesis genes, and **c** regulatory and transporter genes of the A and C genomes, determined using qRT-PCR with samples of the 4th leaf of 6-week-old plants of Asia (donor of A genome), Rubea (donor of C genome), and the resynthesized line Rs035 (red phenotype), with Rs306 (green phenotype) as a control. Error bars represent the standard deviation of the means of three independent replicates for each genotype, while different letters indicate significant differences among the genotypes at *p * < 0.01, following Tukey’s method

Among the upstream genes, *CHS*, *CHI*, *F3′H,* and *FLS1* showed 2-, 10-, 1.8-, and 20-fold higher levels of expression in the A genome of Rs035 than in its C genome, respectively, and were even 1.5-, 1.8-, 2-, and 10-fold more highly expressed than in the A-genome donor parental line, Asia (Fig. 4a). By contrast, *F3H* gene showed 1.6-fold higher expression in the C-genome of Rs035 than its A-genome. These results indicate that the highly upregulated upstream genes in the A and C genomes of Rs035 could be a major factor in its high accumulation of anthocyanin, although the genes from both the A and C genomes were upregulated compared to the donor parents, indicating a synergistic or positive effect of the two genomes overall. In contrast, the upstream gene *F3H* in the C genome of Rs035 showed a 1.5-fold higher level of expression than that of in A-genome, and a 15-fold higher expression than in the C-genome donor line, Rubea (Fig. 4a). The upstream gene *F3′H* in the A genome of Rs035 had twofold higher expression compared to A-genome donor line, Asia. These results indicate that *F3H* and *F3′H* had a strong positive effect on enhancing the accumulation of anthocyanin accumulation in Rs035, but A and C genomes complemented each other.

The A-genome downstream anthocyanin biosynthesis genes *DFR*, *ANS*, and *UGT75C1* were more highly expressed in Rs035 than their C-genome counterparts with 60-, 4-, and 55-fold higher expression levels, respectively. *DFR*, *ANS*, and *UGT75C1* were expressed 5-, 3-, and 1.5-fold more highly in Rs035, respectively, than in the A-genome donor parent, Asia (Fig. 4b). Twelve biosynthetic genes (*PAL1*, *PAL2*, *4CL1*, *CHS*, *CHI*, *F3H*, *F3′H*, *UGT78D2*, *FLS1*, *DFR*, *ANS*, and *UGT75C1*) showed a transgressive overexpression (significantly exceeding the expression levels of both parental species) in the resynthesized line Rs035 in A genome background (Fig. 4a, b and Additional file 5: Figure S3a). Moreover, eight structural and biosynthetic genes (*PAL1*, *PAL2*, *C4H*, *CHS*, *F3′H*, *FLS1*, *DFR*, and *ANS*) showed higher expression in the resynthesized line Rs035 in C genome background compared to Rs035 in A genome background (Fig. 4a, b and Additional file 5: Figure S3a). The other structural genes of the A and C genomes showed non-additive expression. These results indicate that the highly expressed downstream genes (*DFR*, *ANS*, and *UGT75C1*) might be responsible for the enhanced accumulation of anthocyanin in Rs035, and that the expression of these genes is significantly upregulated by the interaction between the A and C genomes.

#### Expression profiling of regulatory and transporter genes

We next attempted to better understand the molecular mechanisms by which R2R3-MYB, bHLH, and WD40 transcription factors regulate anthocyanin biosynthesis in the line Rs035. To investigate whether any of the regulatory or transporter genes were upregulated to control the expression of the anthocyanin structural genes or affect anthocyanin accumulation, we performed an expression analysis of two independent regulatory R2R3-MYB genes (*MYB12* and *MYB111*), four genes of the MBW complex [the MYBs *PAP2* and *MYB113,* the bHLH *TT8*, and the WD40 *Transparent Testa Glabra 1* (*TTG1*)], and one transporter gene (*TT19*) from both the A and C genomes. Among these seven genes, *MYB12*, *PAP2*, *MYB113*, and *TTG1* from the A and C genome backgrounds of Rs035 did not show any striking difference in expression, although the A genome genes were quantitatively more highly expressed than those of the C genome, and were even higher than in the A genome donor parental line, Asia (Additional file 5: Figure S3b). In contrast, the regulatory gene *MYB111* and the transporter gene *TT19* in A genome background were significantly more highly expressed in Rs035 than *MYB111* and *TT19* in C genome background (170-fold and 75-fold higher expression, respectively). *TT8* of C genome showed a 15-fold higher expression level than *TT8* of A genome in Rs035 (Fig. 4c). In addition, five regulatory and transporter genes, of which four from A genome (*MYB12*, *PAP2*, *MYB111* and *BrTT19*) and one from C genome (*TT8*) showed transgressive overexpression and two genes (*MYB113* and *MYB111*) of C genome showed transgressively repressed expression in the line Rs035, while one regulatory gene (*MYB12*) of C genome showed additive expression in Rs035 (Fig. 4c and Additional file 5: Figure S3b). These results also revealed that the interaction of the A and C genomes led to synergistic or additive effects on gene expression, possibly due to the complementary actions of the syntenic homeologous regulatory and transporter genes.

### Associations between the constituent anthocyanins and the expression levels of the biosynthetic and regulatory genes

**Fig. 5:**
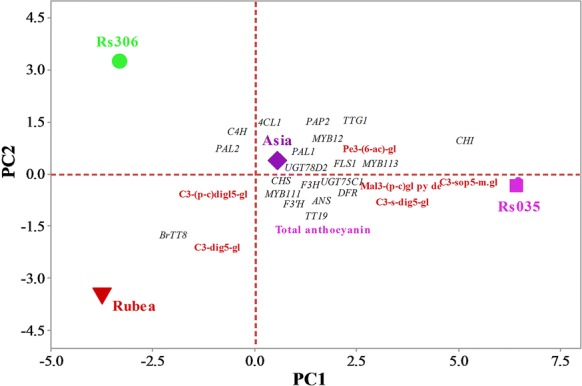
Biplot of total and constituent anthocyanin contents and the expression of anthocyanin biosynthesis pathway genes in four *Brassica* lines (Asia, Rubea, Rs035, and Rs306), determined using a principle component analysis (PCA). Genotype text color reflects the visual phenotype of the leaves. Blue and italic texts denote anthocyanin components and gene names, respectively. *C3-dig5-gl* cyanidin 3-diglucoside-5-glucoside, *C3-s-dig5-gl* cyanidin 3-(sinapoyl)-diglucoside-5-glucoside, *C3-sop5-m.gl* cyanidin 3-sophoroside-5-malonylglucoside, *Mal3-(p-c)gl py de* malvidin-3-(*p*-coumaroyl)glucoside pyruvic derivative, *Pe3-(6-ac)-gl* pelargonidin-3-(6-acetyl)-glucoside, *C3-(p-c)digl5-gl* cyanidin 3-(*p*-coumaroyl) diglucoside-5-glucoside

A principal component analysis of the total and constituent levels of anthocyanins and the expressions of the biosynthesis pathway genes in four contrasting lines (Asia, Rs306, Rs035, and Rubea) led to the identification of five principal components (PCs) with an Eigenvalue greater than unity (data not shown). The first three PCs (PC1, PC2, and PC3) explained 97% of the total variance in anthocyanin content (67.5%, 22.5%, and 7% of the variance was explained by PC1, PC2, and PC3, respectively; Table 2). The total variation accounted for by PC1 was manifested by the higher positive coefficients of some of the upstream and all of the downstream biosynthesis and regulatory genes, the total anthocyanin content, and the majority of the individual anthocyanin components, versus the higher negative coefficients of two upstream biosynthesis genes and one regulatory gene (Table 2 and Fig. 5). PC1 clearly distinguished the red resynthesized line (Rs035) from the green line (Rs306) and the two parental lines (Asia and Rubea). This distinction corresponded to the highly expressed genes, anthocyanin components, and total anthocyanin contents of the four samples, as evidenced by their mean PC scores and different positions in the PCA biplot (Table 2 and Fig. 5). The highly expressed biosynthetic and regulatory genes were plotted against the total anthocyanin content and most of the anthocyanin components in the high-anthocyanin line, Rs035, as shown in the PCA biplot (Fig. 5).

**Table 2:** Component loadings and mean principal component (PC) scores, showing the association between the total and constituent anthocyanin contents and the expression levels of the biosynthetic and regulatory genes, as determined using a principal component analysis

Variable	PC1	PC2	PC3
Upstream genes			
*PAL1*	0.14	0.29	0.24
*PAL2*	− 0.06	0.36	0.26
*C4H*	− 0.09	0.34	0.24
*4CL1*	− 0.04	0.37	0.25
*CHS*	0.22	− 0.01	− 0.19
*CHI*	0.23	0.01	− 0.09
*F3H*	0.21	− 0.05	0.19
*F3´H*	0.23	− 0.08	− 0.03
Downstream genes			
*FLS1*	0.23	0.02	0.11
*DFR*	0.23	− 0.03	0.15
*ANS*	0.23	− 0.08	0.12
*UGT75C1*	0.22	− 0.01	− 0.17
*UGT78D2*	0.23	0.03	0.10
Regulatory genes			
*MYB12*	0.23	0.04	− 0.05
*MYB111*	0.22	− 0.03	− 0.22
*PAP2*	0.22	0.08	0.10
*MYB113*	0.23	0.01	− 0.05
*BrTT8*	− 0.11	− 0.34	0.17
*TTG1*	0.20	0.11	− 0.15
*TT19*	0.22	− 0.03	0.21
Anthocyanin components			
C3-dig5-gl	− 0.04	− 0.38	0.23
C3-s-dig5-gl	0.21	− 0.10	0.28
C3-sop5-m.gl	0.23	− 0.01	− 0.07
Mal3-(p-c)gl py de	0.21	− 0.03	0.28
Pe3-(6-ac)-gl	0.20	0.01	− 0.37
C3-(p-c)digl5-gl	− 0.12	− 0.33	0.15
Total anthocyanin	0.09	− 0.35	0.23
Eigenvalue	18.22	6.07	2.10
% of the variation explained	67.5	22.5	7.0
Cumulative % of the variation explained	67.5	90.0	97.0
Genotype	Mean PC score ± SD		
Asia	0.57 ± 0.09 B	0.36 ± 0.12 B	− 2.37 ± 0.20 C
Rs306	− 3.31 ± 0.02 C	3.28 ± 0.11 A	0.89 ± 0.09 A
Rs035	6.45 ± 0.04 A	− 0.29 ± 0.08 C	0.96 ± 0.08 A
Rubea	− 3.72 ± 0.02 D	− 3.35 ± 0.07 D	0.52 ± 0.04 B

*SD* standard deviation, *C3-dig5-gl* cyanidin 3-diglucoside-5-glucoside, *C3-s-dig5-gl* cyanidin 3-(sinapoyl)-diglucoside-5-glucoside, *C3-sop5-m.gl* cyanidin 3-sophoroside-5-malonylglucoside, *Mal3-(p-c)gl py de* malvidin-3-(*p*-coumaroyl)glucoside pyruvic derivative, *Pe3-(6-ac)-gl* pelargonidin-3-(6-acetyl)-glucoside, *C3-(p-c)digl5-gl* cyanidin 3-(*p*-coumaroyl) diglucoside-5-glucoside

Letters in mean PC score against the genotypes are mean separation using Tukey test with *p *<* 0.05*

Meanwhile, PC2 clearly separated the resynthesized line Rs035 from its diploid parents *B. rapa* (Asia) and *B. oleracea* (Rubea) in the PCA biplot, along with their respective gene expression patterns, total anthocyanin contents, and anthocyanin compositions (Fig. 5). This was further evidenced by the mean PC scores in the resynthesized lines, Rs035 and Rs306 (+ 6.45 and − 3.31, respectively), and the diploid parental lines, Rubea and Asia (− 3.72 and 0.57, respectively; Table 2), and by the higher significant positive correlations between total anthocyanin and the gene expression patterns, as observed from the Pearson’s correlation analysis (Additional file 3: Table S2).

## Discussion

The anthocyanins are a group of flavonoid secondary metabolites with a wide range of colors, from light yellow to blue or even black. *Brassica* vegetables have diverse anthocyanin contents; however, very few high-anthocyanin cultivars are available for *B. napus*. Previously, only one report has investigated the quantitative trait loci regulating anthocyanin accumulation in this species [14]. Here, we attempted to develop resynthesized *B. napus* lines with enhanced levels of anthocyanin in the leaves by hybridizing high-anthocyanin varieties of *B. rapa* var. *pekinensis* (A genome) and *B. oleracea* var. *capitata* (C genome) followed by chromosome doubling. Many interspecific hybrids have been developed from various wild and cultivated A and C genome plants [40]; however, this is the first report of the hybridization of two anthocyanin-enriched diploid *Brassica* species. A total of 39 tetraploid hybrids with highly variable total anthocyanin contents were generated (Fig. 2), suggesting recombination between homoeologous chromosomes, and/or altered DNA methylation status of the genes, which could lead to rapid phenotypic changes in the newly resynthesized allopolyploids [39, 41–47]. Several studies on resynthesized polyploids (including in the genera *Arabidopsis, Brassica, Gossypium, Nicotiana, Triticum*, and *Triticale*) and natural polyploid species have demonstrated a variety of genomic changes, including deletions, gene conversions, transposon activations, chromosomal rearrangements, and epigenetic modifications, all of which altered the phenotypes of the resulting plants [48–50].

By using HPLC, we identified five anthocyanin compounds in Rs035, whereas only four and three compounds were detected in Asia and Rubea, respectively (Table 1). The additional anthocyanin compound in Rs035 might be the result of recombination and/or a genomic restructuring of the anthocyanin biosynthetic and regulatory genes between its A and C genomes. The genomes of allopolyploids can be considerably different from those of the parental genomes [51–53].

We identified 73 and 58 genes involved in the anthocyanin pathway in *B. rapa* (A genome) and *B. oleracea* (C genome), respectively. The A-genome genes were distributed across all 10 A-genome chromosomes (A01–A10) and the C-genome genes were located on all nine C-genome chromosomes (C01–C09). Some of the genes could not be mapped to chromosomes and were instead mapped to scaffolds; more C-genome than A-genome genes were mapped to these non-contiguous genomic sequences (Additional file 1: Table S1a, Additional file 2: Table S2b and Additional file 5: Figure S2a, b). Similar distributions of anthocyanin genes were previously reported [28], and whole-genome analyses established that the degree of gene density on the chromosomes might be the result of genome triplication events in *Brassica* [54, 55]. The genomes of *B. rapa* and *B. oleracea* therefore contain more than one copy of most anthocyanin biosynthesis genes. The microsynteny analyses among the anthocyanin biosynthesis and regulatory genes in *A. thaliana*, *B. rapa*, and *B. oleracea* corroborated the close relationships among them, and confirmed their evolution from a common ancestor, consistent with the report on *BrAQP* genes by Kayum et al. [56].

Anthocyanins are important secondary metabolites that play a defensive role against a range of abiotic and biotic stresses in plants. Recent studies have also reported multiple benefits of anthocyanin consumption in human health. Despite their importance, the genes involved in anthocyanin biosynthesis and its regulation are poorly understood in *B. napus*, although they have been well characterized in other Brassicaceae species, including Arabidopsis [57], Chinese cabbage [29], cabbage [11], pak choi (*B. rapa* ssp. *chinensis*) [58], kohlrabi [27], radish (*Raphanus raphanistrum* ssp. *sativus*) [59], and brown mustard (*B. juncea*) [60]. Here, we tried to predict the putative candidate gene(s) involved in anthocyanin biosynthesis, using expression profiling to assess the magnitude and nature of their expression patterns. Previous studies reported that MBW transcriptional activation complexes may activate the expression of the anthocyanin biosynthesis genes [23, 25, 61]. Our expression data showed that the biosynthesis genes *PAL*, *C4H*, *4CL*, *CHS*, *CHI*, *F3H*, and *F3′H* were differentially expressed among the two parental lines (Asia and Rubea) and the two resynthesized lines (green Rs306 and red Rs035).

Among the upstream genes, A-genome *CHS* and *CHI* showed 2- and tenfold higher levels of expression, respectively, in Rs035 than the C-genome versions of these genes, while only one C-genome upstream gene, *F3H*, showed a 1.5-fold higher expression than the A-genome gene (Fig. 4a). The highly expressed upstream genes seem to be putatively involved in the production of flavonols and other flavonoid compounds in the resynthesized line Rs035. Of the downstream genes, *FLS1*, *DFR*, *ANS*, and *UGT75C1* from the A- and C-genomes were differentially expressed in the examined lines; A-genome *FLS1*, *DFR*, *BrANS*, and *UGT75C1* had 20-, 60-, 4-, and 55-fold higher expression levels, respectively, in Rs035 than their C-genome counterparts. Highly expressed genes downstream of the anthocyanin biosynthesis pathway may contribute to the accumulation of anthocyanin [62].

Seven upstream (*PAL1*, *PAL2*, *4CL1*, *CHS*, *CHI*, *F3H*, *F3′H*) and four downstream genes (*FLS1*, *DFR*, *ANS,* and *UGT75C1*) showed transgressive overexpression in Rs035 in comparison with its parents. Most of the upstream and downstream genes of the Rs035 C-genome showed additive expression (Fig. 4a, b and Additional file 5: Figure S3a). The transgressive overexpression of the above-mentioned genes might be caused by several mechanisms encountered in the hybrid [63], including the reunion of divergent genomes in the same nucleus, enabling the interaction of divergent genes [64] or a dosage effect of the homeologous genes within the same background.

Based on their significantly higher expression levels in one or both genomes of Rs035 in comparison with the parental lines, we predicted that one R2R3-MYB (*MYB111*) and one bHLH (*TT8*) of the MBW complex, as well as a transporter gene (*TT19*), may be responsible for the higher anthocyanin accumulation in Rs035 (Fig. 4c and Additional file 5: Figure S3b). High anthocyanin accumulation in Rs035 might achieved due to combined and complementary effect of those three regulatory and transporter genes by activating the transcription of structural genes, while one regulatory R2R3-MYB (*MYB111*) as well one transporter gene (*TT19*) were upregulated in the parent line Asia; one bHLH (*TT8*) of the MBW complex was upregulated in the parent Rubea. The combined effect of those regulatory and transporter genes might have higher effect on activating the transcription of structural genes in Rs035 than in their parents.

The R2R3-MYB *PAP1*, *MYB113*, and *MYB114*, and the bHLH *TT8* are known to be involved in the upregulation of anthocyanin biosynthesis in Arabidopsis leaves [65]. Xie et al. [29] reported that the bHLH-encoding regulatory genes and a large proportion of structural genes are candidates for the accumulation of anthocyanins in *B. rapa*. In *A. thaliana* and a number of other species, MBW complexes were identified as the activators of anthocyanin biosynthesis [66, 67]. In the resynthesized line Rs035, R2R3-MYB and bHLH transcription factors could bind to specific *cis*-elements in the promoters of the anthocyanin structural genes; this finding is in agreement with [26]. The transgressive expression of the predicted regulatory and transporter genes in the resynthesized line Rs035 might also be due to interaction of *cis*- and *trans*-regulatory factors in the synthetic hybrids as reported by other research efforts [68–70].

Based on our comparative univariate expression data, we identified three early-biosynthesis (*CHS*, *CHI*, and *F3H*), four late-biosynthesis (*FLS1*, *DFR*, *ANS*, and *UGT75C1*), two transcriptional regulator (*MYB111* and *TT8*), and one transporter (*TT19*) genes as the putative candidates responsible for the enhanced accumulation of anthocyanins in the resynthesized line Rs035 (Fig. 4a–c and Additional file 5: Figure S3a, b). We deployed a multivariate analytical approach to identify and visualize the overall association of the expression of these genes with the total and constituent anthocyanin contents in the four *Brassica* lines. The association of the highly expressed genes with the total anthocyanin contents and the anthocyanin components of Rs035 in the PCA biplot indicate that these genes may be responsible for the higher accumulation of anthocyanins in this line. The total anthocyanin contents and the expression of key regulators (*MYB111*, *TT8*, and *TT19*) showed significant positive correlations with the expression levels of the structural genes, which further demonstrated the association between these genes and the total anthocyanin contents. Previous studies indicated that each *B. rapa* and *B. oleracea* chromosome has a homologous counterpart in *B. napus* [71, 72]; therefore, the predicted A- and C-genome genes are likely confined within the resynthesized *B. napus*. A previous study reported the upregulation of *BrCHS*, *BrF3H*, *BrANS*, *BrDFR*, and *BrTT8* in anthocyanin-enriched pak choi [27], while *BoF3′H*, *BoMYB2*, and *BoTT8* were hypothesized to be responsible for anthocyanin accumulation in red cabbage [11]. Our PCA biplot data support the involvement of these genes in the accumulation of high levels of anthocyanin in Rs035 (Fig. 5).

## Conclusions

Anthocyanin-enriched *B. napus* lines were developed by hybridizing anthocyanin-rich varieties of *B. rapa* and *B. oleracea*. A series of in silico analyses were used to identify and characterize the anthocyanin genes and determine their chromosomal distributions and syntenic relationships. Variable numbers of anthocyanin genes are distributed across all chromosomes of the reconstituted *B. napus* lines, which were inherited from the A- and C-genome donor parents. Anthocyanin biosynthesis is a complex enzymatic process; therefore, a coordinated analysis of the expression of many anthocyanin biosynthesis-related genes was carried out to elucidate the molecular mechanisms involved, within the limits of the environmental cues that control the pathway and the final accumulation of the end products. An overview of the most highly expressed genes of each of the pathway nodes in our study revealed that the late-biosynthesis genes of the A-genome are comparatively highly expressed in Rs035 line compared with those of the C genome in the same background, but A and C genomes complemented each other. Our expression data also clearly demonstrated that the independent regulator *BrMYB111*, *BolTT8*, and the transporter gene *BrTT19* are key regulators controlling the production of high levels of anthocyanin in resynthesized *B. napus*, as demonstrated by their transgressive and additive expression patterns. Our study lays the foundation for enabling the development of high-anthocyanin-accumulating cultivars of *B. napus*, with brilliant colors and health-promoting benefits that may be attractive to both growers and consumers. In addition, our resynthesized lines and the differentially expressed genes we have identified could be used to transfer the anthocyanin traits to other commercial rapeseed lines using molecular and conventional breeding.

## Methods

### Plant materials and sampling

Four lines from the genus *Brassica* were used, including a red-green Chinese cabbage line (*B. rapa* cv. Asia), a red cabbage line (*B. oleracea* cv. Rubea), a green resynthesized rapeseed line (allopolyploid *B. napus* cv. Rs306), and a red resynthesized rapeseed line (allopolyploid *B. napus* cv. Rs035). The Rs035 line was developed by crossing the inbred Chinese cabbage line *B. rapa* cv. Asia with the cabbage line *B. oleracea* cv. Rubea. The embryos were rescued 18–20 days after crossing and were cultured on Murashige and Skoog (MS) medium. The chromosomes of the developed plants were diploidized using a 4-hr 0.1% colchicine root treatment. Successfully crossed hybrid plants were identified using the conserved ortholog set (COS) marker COS1078 [73]. The genotypes of the allopolyploid plants with diploid chromosomes were confirmed using a CyFlow ploidy analyzer and 4′,6-diamidino-2-phenylindole (DAPI) solution (Sysmex, Norderstedt, Germany).

### RNA extraction and cDNA synthesis

The fourth leaves of 6-week-old plants were collected and immediately frozen in liquid nitrogen, then stored at − 80 °C. Around 2 g of each leaf sample was ground into powder for RNA extraction and anthocyanin quantification. Total RNA was isolated from 100 mg of the finely ground powder using a RNeasy Plant Mini Kit (Qiagen, Hilden, Germany), according to the manufacturer’s instructions. The RNA was treated with RNase-free DNase I (Qiagen) before cDNA synthesis. The concentration of the extracted RNA was measured using a NanoDrop ND-1000 spectrophotometer (Thermo Fisher Scientific, Waltham, MA, USA). The cDNA was synthesized using a First-Strand cDNA Synthesis kit (Thermo Fisher Scientific) in a 20-μl reaction, according to the manufacturer’s instructions, then stored at − 20 °C until required.

### Quantification of total anthocyanin content

The total anthocyanin content was determined following a previously described protocol [74] with some modifications. Three leaves of each genotype were frozen in liquid nitrogen and ground into powder, then 100 mg of each sample was transferred into an Eppendorf tube containing 1 ml acidic methanol (1% HCl, w/v). Samples were mixed overnight at room temperature by shaking at 50 rpm in the dark. After that, the mixtures were centrifuged at 12,000×*g* for 10 min. The supernatants were collected and the absorbance of each sample was determined at 530 and 657 nm wavelengths. The total anthocyanin content was computed using the following equation: Q_Anthocyanins_ = (A_530_ – 0.25 × A_657_) × FW^−1^, where Q_Anthocyanins_ = total anthocyanin content, A_530_ = absorption at 530 nm, A_657_ = absorption at 657 nm, and FW = fresh weight of leaf samples (g). The total anthocyanins were quantified from five replicates of each biological sample.

### Extraction and quantification of anthocyanin compounds using high-performance liquid chromatography

For the high-performance liquid chromatography (HPLC) analysis, the leaf samples were freeze-dried and ground into a fine powder in liquid nitrogen. A 100-mg sample of each finely powdered leaf was vortexed for 5 min in 2 ml 5% formic acid (v/v) in ultrapure water, after which it was sonicated for 20 min. The samples were centrifuged at 12,000 rpm for 10 min, then the supernatant was filtered through a 0.45-μm PTFE filter (Toyo Roshi Kaisha, Ltd., Tokyo, Japan). The extracted anthocyanins were quantified using an Agilent 1200 series HPLC (Agilent Technologies, Santa Clara, CA, USA), and the chromatographic separation was carried out using reverse phase columns (particle size 4 μm; C18 80A, 250 × 4.60 mm; Phenomenex, Torrence, CA, USA) at a temperature of 40 °C. The injection volume was 10 μl with a flow rate of 1 ml min^−1^. Elution was performed using mobile phase A (5% formic acid) and B (acetonitrile), with the following gradient program: 0–8 min using 5–10% B; 8–15 min at 13% B; 15–18 min using 15% B and kept constant until 25 min. After 25 min, the concentration of B was quickly decreased to 5% and kept constant until 35 min. Detection was carried out at a wavelength of 520 nm. Individual anthocyanin components were quantified by comparing the area under their HPLC peak with that of a known standard (cyanidin-3-0-glucoside), and expressed as mg g^−1^ dry weight.

### Selection and in silico analysis of anthocyanin biosynthesis genes

The anthocyanin pathway genes of the A and C genomes were collected from the BRAD database (http://brassicadb.org/brad/) using a syntenic gene search of the anthocyanin pathway genes in *A. thaliana* retrieved from TAIR (https://www.arabidopsis.org/). These genes were also checked against the Bolbase (http://www.ocri-genomics.org/bolbase/genes.htm) and EnsemblPlants (http://plants.ensembl.org/) databases. Finally, a complementary method, Hidden Markov Models profiling, was performed to increase the accuracy of the gene identification in the *B. rapa* and *B. oleracea* genomes. In addition, the primary gene features (gene structure, distribution on chromosomes, gene position, strand, protein length, molecular weight, and iso-electric point) were analyzed using ExPasy (http://au.expasy.org/tools/pi_tool.html). The physical positions of the genes were drafted onto the A- and C-genome chromosomes from *B. rapa* and *B. oleracea*, respectively, using Map Chart v.2.2 (http://www.wageningenur.nl/en/show/Mapchart.htm). The microsyntenic relationships of the anthocyanin structural, regulatory, and transporter genes in *A. thaliana, B. rapa*, and *B. oleracea* were plotted using circos software (http://circos.ca/) [75].

### Expression analysis using quantitative real-time (qRT) PCR

Gene expression levels were analyzed using qRT-PCR performed on a Roche LightCycler^®^ 96 System (Roche Applied Science, Penzberg, Germany). Gene-specific primers (Additional file 4: Table S3a, b) were designed using an online tool, Primer3Plus [76], and were targeted to the gene sequences collected from BRAD [77] and Bolbase [55]. The 10-μl reaction mixture contained 5 μl of 2 × qPCRBIO SyGreen Mix Lo-ROX (PCR Biosystems, London, UK), 1 μl of each gene-specific forward (F) and reverse (Rv) primer (10 pmol), 2 μl distilled deionized water (ddH_2_O), and 1 μl of 80 ng μl^−1^ cDNA as a template. The qRT-PCR was performed under the following conditions: initial denaturation at 95 °C for 5 min; followed by 50 cycles of 95 °C for 10 s, 58 °C for 10 s, and 72 °C for 15 s. Three replicates of each sample were analyzed. The data were processed using the LightCycler 96 SW 1.1 software and quantified using the Cq value of each qRT-PCR amplicon, following the 2^−ΔΔCt^ method [78]. The data were normalized against the mean amplicon value of the three *ACTIN* genes (as internal controls) expressed by *B. rapa* and *B. oleracea*.

### Statistical analysis

The total and constituent anthocyanin contents and the gene expression levels were analyzed using one-way analysis of variance (ANOVA) and Tukey’s pair-wise comparisons in Minitab v.17 (Minitab Inc., State College, PA, USA). The data were presented as the mean of three replicates ± standard deviation (± SD). Data used for principal component analyses (PCAs) were standardized by subtracting the mean and dividing the result by the standard deviation. The constituent anthocyanins determined using HPLC and the relative gene expression levels calculated from the qRT-PCR were set as variables for the PCA using Minitab v.17 (Minitab Inc.).

## Additional files

**Additional file 1: Table S1a.** In silico analysis of Anthocyanin genes identified in *B. rapa* with their Arabidopsis orthologs and biological activity.

**Additional file 2: Table S1b.** In silico analysis of anthocyanin genes identified in *B. oleracea* with their Arabidopsis orthologs and biological activity.

**Additional file 3: Table S2.** Pearson’s correlation analysis among the expression of the anthocyanin biosynthesis pathway genes of A- and C-genome along with anthocyanin components in the four contrasting Brassica lines.

**Additional file 4: Table S3a.** Oligonucleotide primers used for qRT–PCR analysis of *B. rapa.* **Table S3b.** Oligonucleotide primers used for qRT–PCR analysis of *B. oleracea*.

**Additional file 5: Figure S1.** Morphological distinctiveness and anthocyanin contents of four *Brassica* lines. The visual phenotype (**a**) and 4th-leaf total anthocyanin contents of 6-week-old plants (**b**) of the parental lines Asia (*B. rapa*) and Rubea (*B. oleracea*), the red allopolyploid resynthesized *B. napus* (Rs035), and the control green allopolyploid resynthesized *B. napus* (Rs306) are shown. **Figure S2.** Chromosomal distributions of the putative anthocyanin biosynthesis genes in (**a**) *Brassica rapa* and (**b**) *Brassica oleracea*. The chromosome number is indicated at the top of each chromosome. The scale (left) is in megabases (Mb). The colors represent the putative structural genes of the phenylpropanoid pathway (black), early-biosynthesis (red), late-biosynthesis (green), positive-regulatory (blue), and negative-regulatory (brown) genes. Genes assigned to scaffold sequences are not shown on the physical maps. **Figure S3.** Relative gene expression of anthocyanin pathway genes which did not show striking expression changed compared among the studied four lines, **(a)** structural and **(b)** regulatory and transporter genes.

## Abbreviations

4CL: 4-coumaratel-coA ligase
ANOVA: analysis of variance
ANS: anthocyanidin synthase
bHLH: basic helix loop helix
Bolbase: *Brassica oleracea* Genomics Database
BRAD: Brassica Database
C4H: cinnamate-4-hydroxylase
cDNA: complementary DNA
CHI: chalcone isomerase
CHS: chalcone synthase
COS: conserved ortholog set
DFR: dihydroflavanol reductase
F3H: flavanone-3-hydroxylase
FLS: flavonol synthase
FW: fresh weight
HPLC: high performance liquid chromatography
MS: Murashige and Skoog
MYB: myeloblastosis family of transcription factors
PAL: phenylalanine ammonia lyase
PCA: principal component analysis
Pi: isoelectric point
qPCR: quantitative polymerase chain reaction
Rs: resynthesised
RT-PCR: reverse transcription polymerase chain reaction
SD: standard deviation
TAIR: the arabidopsis information resource
TT: transparent testa
TTG1: transparent testa glabra 1
UGT: uradin glycosyltransferase
