# Supplementary material for: Transcriptional regulation of anthocyanin biosynthesis in a high-anthocyanin resynthesized Brassica napus cultivar
Source: J Biol Res (Thessalon). 2018 Nov 26;25:19. doi: 10.1186/s40709-018-0090-6 (PMC6258291; doi:10.1186/s40709-018-0090-6)
Supplement: Supplementary file 1 — Additional file 1: Table S1a. In silico analysis of Anthocyanin genes identified in B. rapa with their Arabidopsis orthologs and biological activity. [file 40709_2018_90_MOESM1_ESM.doc]

**Table S1a. *In silico* analysis of Anthocyanin genes identified in *B. rapa* with their Arabidopsis orthologs and biological activity**

| Gene name | Gene ID | Chromosome | | | Strand | Sub-genome | Isoelectric point (Pi) | Molecular  weight  (Dalton) | Protein  Length (aa) | Orthologous  gene | Biochemical activity |
| --- | --- | --- | --- | --- | --- | --- | --- | --- | --- | --- | --- |
|  |  | No. | Start | End |  |  |  |  |  |  |  |
| *BrPAL1.1* | Bra005221 | A05 | 4098130 | 4100879 | + | LF | 5.98 | 40480.46 | 371 | [AT2G37040](javascript:modalDialog('multiSearchAt.php?gene=AT2G37040','select database',390,200)) | Phenylalanine ammonia-lyase 1 |
| *BrPAL1.2* | Bra017210 | A04 | 16132008 | 16134574 | **-** | MF1 | 5.90 | 78297.28 | 721 | [AT2G37040](javascript:modalDialog('multiSearchAt.php?gene=AT2G37040','select database',390,200)) | Phenylalanine ammonia-lyase 1 |
| *BrPAL2.1* | [Bra006985](javascript:modalDialog('multiSearch.php?gene=Bra006985','select database',390,200)) | A09 | 28184757 | 28187233 | - | LF | 6.16 | 76619.38 | 706 | [AT3G53260](javascript:modalDialog('multiSearchAt.php?gene=AT3G53260','select database',390,200)) | Phenylalanine ammonia-lyase 2 |
| *BrPAL2.2* | [Bra039777](javascript:modalDialog('multiSearch.php?gene=Bra039777','select database',390,200)) | A04 | 3961512 | 3964566 | + | MF1 | 4.94 | 126182.18 | 1533 | [AT3G53260](javascript:modalDialog('multiSearchAt.php?gene=AT3G53260','select database',390,200)) | Phenylalanine ammonia-lyase 2 |
| *BrPAL2.3* | Bra003126 | A07 | 14754085 | 14756786 | - | MF2 | 4.90 | 173751.10 | 2124 | [AT3G53260](javascript:modalDialog('multiSearchAt.php?gene=AT3G53260','select database',390,200)) | Phenylalanine ammonia-lyase 2 |
| *BrPAL3.1* | [Bra028793](javascript:modalDialog('multiSearch.php?gene=Bra028793','select database',390,200)) | A02 | 625387 | 626594 | + | MF2 | 6.20 | 32558.57 | 294 | [AT5G04230](javascript:modalDialog('multiSearchAt.php?gene=AT5G04230','select database',390,200)) | Phenylalanine ammonia-lyase 3 |
| *BrPAL3.2* | Bra030322 | A04 | 9481038 | 9483492 | - | MF1 | 6.07 | 76922.83 | 698 | AT5G04230 | Phenylalanine ammonia-lyase 3 |
| *BrPAL4* | Bra029831 | A05 | 22819640 | 22826303 | - | LF | 5.69 | 76880.67 | 706 | AT3G10340 | Phenylalanine ammonia-lyase 4 |
| *BrC4H1* | Bra018311 | A05 | 7378464 | 7380483 | + | LF | 9.04 | 57935.36 | 505 | AT2G30490 | Trans-cinnamate 4-monooxygenase 3 |
| *BrC4H2* | Bra021636 | A04 | 13681735 | 13687441 | - | MF1 | 9.17 | 57481.84 | 505 | AT2G30490 | Trans-cinnamate 4-monooxygenase 3 |
| *BrC4H3* | Bra021637 | A04 | 13688684 | 13690602 | - | MF1 | 9.14 | 57829.16 | 505 | AT2G30490 | Trans-cinnamate 4-monooxygenase 3 |
| *BrC4H4* | Bra022802 | A03 | 7147092 | 7149367 | - | MF2 | 8.89 | 57551.02 | 503 | AT2G30490 | Trans-cinnamate 4-monooxygenase 3 |
| *BrC4H5* | Bra022803 | A03 | 7151974 | 7153871 | - | MF2 | 9.06 | 57695.06 | 505 | AT2G30490 | Trans-cinnamate 4-monooxygenase 3 |
| *Br4CL1* | Bra030429 | A05 | 11246278 | 11249002 | + | MF2 | 5.38 | 60085.35 | 552 | AT1G51680 | 4-coumarate:CoA ligase 1 |
| *Br4CL2.1* | Bra031262 | A05 | 17306583 | 17311892 | + | LF | 5.49 | 69810.57 | 609 | AT3G21240 | 4-coumarate:CoA ligase 2 |
| *Br4CL2.2* | Bra031263 | A05 | 17296799 | 17300456 | + | LF | 8.79 | 60811.63 | 553 | AT3G21240 | 4-coumarate:CoA ligase 2 |
| *Br4CL2.3* | Bra031265 | A05 | 17276121 | 17280811 | + | LF | 5.65 | 61117.85 | 558 | AT3G21240 | 4-coumarate:CoA ligase 2 |
| *Br4CL2.4* | Bra031266 | A05 | 17255035 | 17255035 | + | LF | 5.73 | 60003.10 | 552 | AT3G21240 | 4-coumarate:CoA ligase 2 |
| *Br4CL3* | Bra004109 | A07 | 20172546 | 20177374 | + | LF | 5.66 | 60441.43 | 556 | AT1G65060 | 4-coumarate:CoA ligase 3 |
| *Br4CL5.1* | Bra001819 | A03 | 18663880 | 18669799 | - | MF2 | 5.26 | 63108.80 | 574 | AT3G21230 | 4-coumarate:CoA ligase 5 |
| *Br4CL5.2* | Bra001820 | A03 | 18675088 | 18678049 | - | MF2 | 5.35 | 64123.86 | 585 | AT3G21230 | 4-coumarate:CoA ligase 5 |
| *BrCHS1* | Bra008792 | A10 | 12655973 | 12657235 | - | LF | 5.92 | 42982.48 | 395 | AT5G13930 | Chalcone synthase 1 |
| *BrCHS2* | Bra006224 | A03 | 2596137 | 2597594 | + | MF1 | 6.57 | 43239.97 | 396 | AT5G13930 | Chalcone synthase 3 |
| *BrCHS3* | Bra023441 | A02 | 2357734 | 2359185 | + | MF2 | 6.61 | 42952.67 | 394 | AT5G13930 | Chalcone synthase 3 |
| *BrCHS4* | Bra036307 | A09 | 695971 | 697415 | + | Non-synteny ortholog | 6.47 | 43179.01 | 393 | AT5G13930 | Chalcone synthase 3 |
| *BrCHS5* | Bra020688 | A02 | 23204660 | 23205928 | + | Non-synteny ortholog | 5.86 | 43019.87 | 394 | AT5G13930 | Chalcone synthase 3 |
| *BrCHI1* | Bra007142 | A09 | 29055564 | 29057157 | - | LF | 4.78 | 27046.92 | 251 | AT3G55120 | Chalcone-flavonone isomerase |
| *BrCHI2* | Bra003209 | A07 | 15173091 | 15175119 | - | MF2 | 6.18 | 24607.31 | 227 | AT3G55120 | Chalcone-flavonone isomerase |
| *BrCHI3* | Bra017728 | A03 | 30108206 | 30109397 | + | Non-synteny ortholog | 5.61 | 21428.92 | 197 | AT3G55120 | Chalcone-flavonone isomerase |
| *BrF3H1* | Bra036828 | A09 | 27095567 | 27097080 | + | LF | 5.45 | 40104.60 | 358 | AT3G51240 | Naringenin,2-oxoglutarate 3-dioxygenase |
| *BrF3H2* | Bra029996 | A01 | 15030232 | 15032156 | + | MF1 | 5.59 | 41416.54 | 364 | AT3G51240 | Deacetoxyvindoline 4-hydroxylase |
| *BrF3H3* | Bra012862 | A03 | 21908585 | 21910045 | + | MF2 | 5.64 | 38901.37 | 350 | AT3G51240 | Naringenin,2-oxoglutarate 3-dioxygenase |
| *BrF3´H* | Bra009312 | A10 | 14356094 | 14358845 |  | LF | 7.31 | 56631.42 | 511 | AT5G07990 | Flavonoid 3'-monooxygenase |
| *BrFLS1* | Bra009358 | A10 | 14152996 | 14154560 | - | LF | 5.48 | 38259.72 | 336 | AT5G08640 | Flavonol synthase 1 |
| *BrFLS2* | Bra038647 | A06 | 14983444 | 14986024 | + | LF | 5.67 | 35305.24 | 307 | AT5G63580 | Flavonol synthase 2 |
| *BrFLS3.1* | Bra038648 | A06 | 14996259 | 14997537 | - | LF | 6.18 | 34406.44 | 297 | AT5G63590 | Flavonol synthase 3 |
| *BrFLS3.2* | Bra029211 | A02 | 25978824 | 25980695 | - | MF1 | 5.33 | 35848.84 | 310 | AT5G63590 | Flavonol synthase 3 |
| *BrFLS3.3* | Bra029212 | A02 | 25974752 | 25976735 | - | MF1 | 6.82 | 48117.34 | 422 | AT5G63590 | Flavonol synthase 3 |
| *BrFLS4* | Bra037747 | A09 | 3988206 | 3991271 | - | MF2 | 6.33 | 35921.95 | 314 | AT5G63595 | Flavonol synthase 4 |
| *BrDFR* | Bra027457 | A09 | 10926334 | 10927890 | - | MF2 | 5.54 | 42886.04 | 385 | AT5G42800 | Dihydroflavonol-4-reductase |
| *BrANS1* | Bra013652 | A01 | 6885692 | 6887113 | - | LF | 5.24 | 40876.07 | 358 | AT4G22880 | Leucoanthocyanidin dioxygenase |
| *BrANS2* | Bra019350 | A03 | 24796232 | 24797395 | - | MF1 | 5.13 | 40774.77 | 357 | AT4G22880 | Leucoanthocyanidin dioxygenase |
| *BrUGT79B1.1* | Bra003021 | A10 | 6063162 | 6064651 | - | LF | 6.37 | 23293.91 | 211 | AT5G54060 | Anthocyanidin 3-O- glucosyltransferase |
| *BrUGT79B1.2* | Bra035004 | Scaffold000100 | 797862 | 799308 | - | Non-synteny ortholog | 5.96 | 23322.91 | 211 | AT5G54060 | Anthocyanidin 3-O-glucosyltransferase |
| *BrUGT75C1* | Bra038445 | A08 | 8755970 | 8757334 | - | MF2 | 5.43 | 50045.70 | 454 | AT4G14090 | Anthocyanin 5-O-glucosyltransferase |
| *BrUGT78D2* | Bra023594 | A02 | 3087246 | 3088800 | - | MF2 | 5.09 | 44741.04 | 409 | AT5G17050 | Flavonoid 3-O-glucosyltransferase |
| *BrMYB12.1* | Bra004456 | A05 | 222923 | 225991 | - | LF | 4.85 | 40446.69 | 365 | AT2G47460 | Transcription factor MYB12 |
| *BrMYB12.2* | Bra000453 | A03 | 11210760 | 11213776 | + | MF2 | 5.34 | 42144.81 | 376 | AT2G47460 | Transcription factor MYB12 |
| *BrMYB111.1* | Bra037419 | A06 | 19963815 | 19966634 | + | LF | 5.02 | 38762.10 | 343 | AT5G49330 | Production of flavonol glycosides 3 |
| *BrMYB111.2* | Bra020647 | A02 | 23411279 | 23411752 | - | MF1 | 3.80 | 17477.82 | 157 | AT5G49330 | MYB domain protein 111; DNA binding / transcription factor |
| *BrMYB111.3* | Bra036145 | A09 | 2246093 | 2248814 | - | MF2 | 5.14 | 39519.77 | 347 | AT5G49330 | Production of flavonol glycosides 3 |
| *BrPAP1(MYB75)* | Bra001917^b^ | A03 | 8839008 | 8840737 | + | Non-synteny ortholog | 9.08 | 28389.59 | 249 | AT1G56650 | Suc-induced anthocyanin accumulation 1 |
| *BrMYB113* | Bra039763^b^ | A02 | 8839008 | 8840737 | + | MF1 | 9.00 | 28459.44 | 249 | AT1G66370 | Suc-induced anthocyanin accumulation 1 |
| *BrMYB113* | Bra004162^b^ | A07 | 20426416 | 20431671 | + | LF | 8.86 | 27946.70 | 247 | AT1G66370 | Production of anthocyanin pigment 2 |
| *BrTT8* | Bra037887 | A09 | 15769736 | 15773288 | + | LF | 5.54 | 57533.12 | 497 | AT4G09820 | Transcription factor TT8 |
| *BrGL3* | Bra25508 | A04 | 8626979 | 8631281 | + | LF | 6.10 | 69729.67 | 629 | AT5G41315 | Glabrous 3 |
| *BrEGL3.1* | Bra027796 | A09 | 6529998 | 6533684 | + | MF1 | 5.14 | 67769.07 | 606 | AT1G63650 | Enhancer of Glabrous 3 |
| *BrEGL3.2* | Bra027653 | A09 | 7531679 | 7534777 | - | MF2 | 5.47 | 66284.38 | 596 | AT1G63650 | Enhancer of Glabrous 3 |
| *BrTTG1.1* | Bra009770 | A06 | 17739539 | 17740552 | - | LF | 4.66 | 37277.91 | 337 | AT5G24520 | Protein transparent testa glabra 1 |
| *BrTTG1.2* | Bra029411 | A02 | 24761813 | 24762175 | - | MF1 | 5.74 | 13455.40 | 120 | AT5G24520 | Protein transparent testa glabra 1 |
| *BrMYBL2.1* | Bra016164 | A07 | 22386380 | 22387236 | - | LF | 9.34 | 21976.83 | 196 | AT1G71030 | MYB-Like 2 |
| *BrMYBL2.2* | Bra007957 | A02 | 11125340 | 11126192 | - | MF1 | 8.61 | 21718.57 | 191 | AT1G71030 | MYB-Like 2 |
| *BrCPC1* | Bra004539 | A05 | 706175 | 706817 | + | LF1 | 9.57 | 10308.84 | 85 | AT2G46410 | CAPRICE |
| *BrCPC2* | Bra039283 | A04 | 18882042 | 18882818 | - | MF1 | 9.46 | 10280.82 | 85 | AT2G46410 | CAPRICE |
| *BrLBD37.1* | Bra012164 | A07 | 11864765 | 11865628 | + | LF | 8.40 | 27868.24 | 256 | AT5G67420 | LOB Domain-containing Protein 37 |
| *BrLBD37.2* | Bra031833 | A02 | 26735230 | 26736023 | + | MF1 | 8.46 | 25985.18 | 241 | AT5G67420 | LOB Domain-containing Protein 37 |
| *BrLBD37.3* | Bra037847 | A09 | 4481055 | 4481859 | + | MF2 | 9.03 | 25719.08 | 236 | AT5G67420 | LOB Domain-containing Protein 37 |
| *BrLBD38.1* | Bra036040 | A09 | 26375754 | 26376800 | + | LF | 7.66 | 27041.19 | 248 | AT3G49940 | LOB Domain-containing Protein 38 |
| *BrLBD38.2* | Bra012913 | A03 | 21565949 | 21566747 | + | MF2 | 6.89 | 26607.10 | 241 | AT3G49940 | LOB Domain-containing Protein 38 |
| *BrLBD39.1* | Bra011772 | A01 | 631422 | 632295 | + | LF | 8.14 | 26167.73 | 237 | AT4G37540 | LOB Domain-containing Protein 39 |
| *BrLBD39.2* | Bra017831 | A03 | 30822548 | 30823423 | - | MF1 | 9.10 | 25301.10 | 232 | AT4G37540 | LOB Domain-containing Protein 39 |
| *BrTT19.1* | Bra008570 | A10 | 11677671 | 11678470 | - | LF | 5.79 | 24564.66 | 213 | AT5G17220 | Transparent Testa 19 |
| *BrTT19.2* | Bra023602 | A02 | 3117740 | 3118547 | + | MF2 | 5.79 | 24735.81 | 215 | AT5G17220 | Transparent Testa 19 |

Note: Highlighted genes with same color are the multiple copies of the same gene.
