# Supplementary material for: Transcriptional regulation of anthocyanin biosynthesis in a high-anthocyanin resynthesized Brassica napus cultivar
Source: J Biol Res (Thessalon). 2018 Nov 26;25:19. doi: 10.1186/s40709-018-0090-6 (PMC6258291; doi:10.1186/s40709-018-0090-6)
Supplement: Supplementary file 2 — Additional file 2: Table S1b. In silico analysis of anthocyanin genes identified in B. oleracea with their Arabidopsis orthologs and biological activity. [file 40709_2018_90_MOESM2_ESM.doc]

**Table S1b. *In silico* analysis of anthocyanin genes identified in *B. oleracea* with their Arabidopsis orthologs and biological activity**

| Gene name | Gene ID | Chromosome | | | Strand | Sub-genome | Isoelectric point (Pi) | Molecular  Weight  (Mw) | Protein length (aa) | Orthologous gene | Biochemical activity |
| --- | --- | --- | --- | --- | --- | --- | --- | --- | --- | --- | --- |
|  |  | No. | Start | End |  |  |  |  |  |  |  |
| *BolPAL1.1* | Bol025522 | C04 | 4708518 | 4711327 | - | LF | 5.86 | 78086.12 | 719 | [AT2G37040](javascript:modalDialog('multiSearchAt.php?gene=AT2G37040','select database',390,200)) | Phenylalanine ammonia-lyase 1 |
| *BolPAL1.2* | Bol037689 | C04 | 37110551 | 37113084 | - | MF1 | 5.97 | 78297.33 | 722 | [AT2G37040](javascript:modalDialog('multiSearchAt.php?gene=AT2G37040','select database',390,200)) | Phenylalanine ammonia-lyase 1 |
| *BolPAL2.1* | Bol025102 | C08 | 28313757 | 28316268 | - | LF | 5.97 | 78528.63 | 723 | [AT3G53260](javascript:modalDialog('multiSearchAt.php?gene=AT3G53260','select database',390,200)) | Phenylalanine ammonia-lyase 2 |
| *BolPAL2.2* | Bol041738 | Scaffold000009_P1 | 1839895 | 1842409 | + | MF1 | 6.03 | 78602.82 | 724 | [AT3G53260](javascript:modalDialog('multiSearchAt.php?gene=AT3G53260','select database',390,200)) | Phenylalanine ammonia-lyase 2 |
| *BolPAL2.3* | Bol005411 | C07 | 15514234 | 15517090 | - | MF2 | 5.54 | 78030.75 | 718 | [AT3G53260](javascript:modalDialog('multiSearchAt.php?gene=AT3G53260','select database',390,200)) | Phenylalanine ammonia-lyase 2 |
| *BolPAL3* | Bol005493 | C02 | 1543376 | 1543951 | + | MF2 | 5.78 | 21580.60 | 191 | [AT5G04230](javascript:modalDialog('multiSearchAt.php?gene=AT5G04230','select database',390,200)) | Phenylalanine ammonia-lyase 3 |
| *BolPAL4* | Bol011375 | Scaffold000212 | 835417 | 839669 | + | LF | 5.73 | 76719.46 | 706 | AT3G10340 | Phenylalanine ammonia-lyase 4 |
| *BolC4H1* | Bol033347 | C04 | 32991664 | 32994016 | - | MF1 | 9.03 | 57732.05 | 505 | AT2G30490 | Trans-cinnamate 4-monooxygenase |
| *BolC4H2* | Bol004608 | C03 | 8129930 | 8132091 | - | MF2 | 9.06 | 55381.57 | 481 | AT2G30490 | Trans-cinnamate 4-monooxygenase |
| *Bol4CL1* | Bol031583 | C07 | 22689086 | 22691730 | + | MF2 | 5.46 | 59971.25 | 551 | AT1G51680 | 4-coumarate--CoA ligase 1 |
| *Bol4CL3* | Bol012584 | C07 | 9420762 | 9425971 | - | LF | 5.80 | 60476.57 | 557 | AT1G65060 | 4-coumarate--CoA ligase 3 |
| *Bol4CL5.1* | Bol038389 | C05 | 20103192 | 20107888 | + | LF | 7.63 | 60711.32 | 553 | AT3G21230 | 4-coumarate--CoA ligase 4 |
| *Bol4CL5.2* | Bol003620 | Scaffold000345 | 93465 | 95615 | + | MF2 | 8.71 | 65403.49 | 584 | AT3G21230 | Medium-chain-fatty-acid--CoA ligase |
| *BolCHS1* | Bol043396 | C09 | 35177565 | 35178827 | - | LF | 5.97 | 43110.63 | 395 | AT5G13930 | Chalcone synthase 3 |
| *BolCHS2* | Bol034259 | C03 | 2348551 | 2350039 | + | MF1 | 6.39 | 43239.97 | 396 | AT5G13930 | Chalcone synthase 3 |
| *BolCHS3* | Bol004243 | Scaffold000328 | 233172 | 235833 | + | MF2 | 5.80 | 60618.22 | 519 | AT5G13930 | Aminopeptidases |
| *BolCHI1* | Bol044343 | C08 | 29339626 | 29341225 | - | LF | 4.98 | 26990.90 | 251 | AT3G55120 | Chalcone--flavonone isomerase |
| *BolCHI2* | Bol008652 | C07 | 29262736 | 29264267 | + | MF2 | 6.76 | 27153.29 | 252 | AT3G55120 | Chalcone--flavonone isomerase |
| *BolF3H1* | Bol010585 | Scaffold000220 | 660367 | 661901 | + | LF | 5.45 | 40170.72 | 358 | AT3G51240 | flavanone 3-hydroxylase 1 |
| *BolF3H2* | Bol010514 | Scaffold000221_P2 | 76113 | 76965 | + | MF1 | 5.29 | 26509.94 | 239 | AT3G51240 | flavanone 3-hydroxylase 2 |
| *BolF3H3* | Bol002277 | Scaffold000395 | 51892 | 53281 | - | MF2 | 5.55 | 39085.49 | 350 | AT3G51240 | flavanone 3-hydroxylase 3 |
| *BolF3`H* | Bol043829 | C09 | 37920268 | 37927936 | - | LF | 7.01 | 56636.45 | 511 | AT5G07990 | Flavonoid 3'-monooxygenase |
| *BolFLS1* | Bol043773 | C09 | 37659733 | 37661523 | - | LF | 5.57 | 38229.74 | 336 | AT5G08640 | flavonol synthase 1 |
| *BolFLS2.1* | Bol019127 | Scaffold000133 | 668234 | 668449 | + | LF | 9.34 | 8508.67 | 71 | AT5G63580 | flavonol synthase 2 |
| *BolFLS2.2* | Bol020738 | Scaffold000121_P2 | 686653 | 688385 | - | MF1 | 5.61 | 35538.59 | 309 | AT5G63580 | flavonol synthase 2 |
| *BolFLS2.3* | Bol019125 | Scaffold000133 | 658589 | 660152 | + | MF2 | 5.77 | 33937.64 | 299 | AT5G63580 | flavonol synthase 2 |
| *BolDFR* | Bol035269 | Scaffold000035_P2 | 114402 | 115980 | + | MF2 | 5.61 | 42910.02 | 385 | AT5G42800 | Dihydroflavonol 4-reductase |
| *BolANS1* | Bol014986 | C01 | 9321817 | 9323185 | - | LF | 5.14 | 40861.01 | 358 | AT4G22880 | Leucoanthocyanidin dioxygenase |
| *BolANS2* | Bol042059 | C06 | 42664426 | 42665589 | - | MF1 | 5.40 | 40776.94 | 358 | AT4G22880 | Leucoanthocyanidin dioxygenase |
| *BolUGT79B1* | Bol038805 | C09 | 21913655 | 21914020 | - | LF | 4.82 | 13643.43 | 121 | AT5G54060 | Anthocyanidin 3-O-glucosyltransferase |
| *BolUGT75C1* | Bol027055 | C08 | 11194305 | 11195678 | - | MF2 | 5.20 | 50140.71 | 457 | AT4G14090 | Anthocyanin 5-O-glucosyltransferase |
| *BolUGT78D3* | Bol021317 | C02 | 4070480 | 4072072 | - |  | 5.41 | 50111.04 | 460 | AT5G17030 | Flavonoid 3-O-glucosyltransferase |
| *BolMYB12.1* | Bol001533 | Scaffold000436 | 11177 | 11944 | - | LF | 4.36 | 28040.57 | 255 | AT2G47460 | Transcription factor MYB12 |
| *BolMYB12.2* | Bol002581 | Scaffold000383 | 164640 | 165350 | - | MF1 | 4.21 | 25322.80 | 236 | AT2G47460 | Transcription factor MYB12 |
| *BolMYB12.3* | Bol029626 | C03 | 13972677 | 13975642 | + | MF2 | 5.16 | 42210.84 | 377 | AT2G47460 | Transcription factor MYB12 |
| *BolMYB111.1* | Bol016599 | Scaffold000153 | 219140 | 221270 | - | LF | 4.94 | 38767.12 | 342 | AT5G49330 | MYB domain protein 111 |
| *BolMYB111.2* | Bol033054 | C02 | 41117857 | 41118315 | - | MF1 | 4.21 | 17136.57 | 152 | AT5G49330 | MYB domain protein 111 |
| *BolMYB111.3* | Bol032351 | C09 | 2837513 | 2838037 | - | MF2 | 4.01 | 19552.12 | 174 | AT5G49330 | MYB domain protein 111 |
| *BolMYB113.1* | Bol012528 | C07 | 8976663 | 8977049 | - | LF | 4.58 | 14193.88 | 128 | AT1G66370 | MYB domain protein 113 |
| *BolMYB113.2* | Bol045347 | Scaffold000001_P2 | 2598801 | 2600517 | + | MF1 | 8.89 | 28359.24 | 249 | AT1G66370 | MYB domain protein 113 |
| *BolTT8* | Bol004077 | Scaffold000332 | 320669 | 323408 | - | LF | 5.40 | 59348.28 | 516 | AT4G09820 | Basic helix-loop-helix (bHLH) DNA-binding superfamily protein |
| *BolGL3* | Bol014556 | Scaffold000173 | 667516 | 670783 | + | LF | 6.19 | 69877.84 | 629 | AT5G41315 | Transcription factor glabra 3 |
| *BolEGL3.1* | Bol022614 | Scaffold000106 | 573699 | 578194 | + | MF1 | 5.15 | 67587.98 | 604 | AT1G63650 | Transcription factor EGL1 |
| *BolEGL3.2* | Bol029662 | Scaffold000064 | 49935 | 53036 | - | MF2 | 5.52 | 66409.65 | 597 | AT1G63650 | Transcription factor EGL1 |
| *BolTTG1* | Bol022420 | C06 | 36113317 | 36114330 | + | LF | 4.66 | 37307.94 | 337 | AT5G24520 | Transducin/WD40 repeat-like superfamily protein |
| *BolMYBL2.1* | Bol016164 | C07 | 4498576 | 4499466 | + | LF | 9.44 | 22307.28 | 196 | AT1G71030 | MYB-like 2 |
| *BolMYBL2.2* | Bol034966 | C02 | 14459358 | 14460121 | - | MF1 | 9.00 | 23509.18 | 207 | AT1G71030 | Transcription repressor MYB6 |
| *BolCPC1* | Bol000928 | Scaffold000489 | 75292 | 75934 | - | LF | 9.57 | 10308.84 | 85 | AT2G46410 | Transcription factor CPC |
| *BolCPC2* | Bol021780 | C04 | 40623628 | 40624435 | - | MF1 | 5.37 | 20091.93 | 258 | AT2G46410 | Transcription factor CPC |
| *BolCPC3* | Bol029590 | C03 | 13657256 | 13659558 | - | MF2 | 6.79 | 19513.49 | 169 | AT2G46410 | Transcription factor CPC |
| *BolLBD37.1* | Bol014304 | Scaffold000175_P1 | 396322 | 397315 | + | LF | 8.32 | 13709.35 | 128 | AT5G67420 | LOB domain-containing protein 37 |
| *BolLBD37.2* | Bol008082 | C02 | 42081440 | 42081775 | + | MF1 | 9.42 | 11991.03 | 111 | AT5G67420 | LOB domain-containing protein 37 |
| *BolLBD37.3* | Bol005707 | C08 | 11789 | 12097 | - | MF2 | 10.47 | 11291.46 | 112 | AT5G67420 | LOB domain-containing protein 37 |
| *BolLBD38.1* | Bol007980 | C08 | 26751089 | 26751412 | + | LF | 9.80 | 12014.60 | 107 | AT3G49940 | LOB domain-containing protein 38 |
| *BolLBD38.2* | Bol021982 | C01 | 18238709 | 18239026 | + | MF1 | 9.25 | 12145.65 | 105 | AT3G49940 | LOB domain-containing protein 38 |
| *BolLBD38.3* | Bol016975 | C06 | 37561452 | 37561778 | + | MF2 | 9.29 | 12316.71 | 108 | AT3G49940 | LOB domain-containing protein 38 |
| *BolTT19.1* | Bol019821 | C09 | 32396394 | 32397205 | - | LF | 5.60 | 24497.48 | 213 | AT5G17220 | Glutathione S-transferase |
| *BolTT19.2* | Bol021325 | C02 | 4115803 | 4116603 | + | MF2 | 5.61 | 24837.91 | 215 | AT5G17220 | Glutathione S-transferase |

Note: Highlighted genes with same color are the multiple copies of the same gene.
