# Supplementary material for: Transcriptional regulation of anthocyanin biosynthesis in a high-anthocyanin resynthesized Brassica napus cultivar
Source: J Biol Res (Thessalon). 2018 Nov 26;25:19. doi: 10.1186/s40709-018-0090-6 (PMC6258291; doi:10.1186/s40709-018-0090-6)
Supplement: Supplementary file 4 — Additional file 4: Table S3a. Oligonucleotide primers used for qRT–PCR analysis of B. rapa. Table S3b. Oligonucleotide primers used for qRT–PCR analysis of B. oleracea. [file 40709_2018_90_MOESM4_ESM.doc]

**Table S3a. Oligonucleotide primers used for qRT–PCR analysis of *B. rapa***

| **Gene name** |  | **Primer sequences (5′-----------------3′)** | **Product size (bp)** | **Gene ID/**  **Acc. no.** | **References** |
| --- | --- | --- | --- | --- | --- |
| *BrPAL1* | Fw | GATTTCTGAACGCCGGAATA | 185 | Bra017210 |  |
|  | Rv | AGGGAGGGAAGGAGTGATGT |  |  |  |
| *BrPAL2* | Fw | TGAGCAGCATAACCAAGACG | 185 | Bra003126 |  |
|  | Rv | TCGCCACTTGAGAAACTGTG |  |  |  |
| *BrC4H* | Fw | AGAAGCTGTCCGGGGATTAT | 165 | Bra018311 |  |
|  | Rv | TACGATGGTGGAGTGGTGAA |  |  |  |
| *Br4CL1* | Fw | AGCTCATCGTCACCGAGTCT | 152 | Bra030429 |  |
|  | Rv | CGTTTCCATTTCGGTTGACT |  |  |  |
| *BrCHS* | Fw | GACCTCGTGGTGGTTGAAGT | 179 | Bra023441 |  |
|  | Rv | TTGACGGAAGGACGAAGACC |  |  |  |
| *BrCHI* | Fw | CTCTCTCCCCTAACGGCTCT | 192 | Bra003209 |  |
|  | Rv | TGGCCAACTTATCTCCAAGG |  |  |  |
| *BrF3H* | Fw | TTCTTCGCCTTACCTCCTGA | 198 | Bra036828 |  |
|  | Rv | CGTCACTTTCACCCATCCTT |  |  |  |
| *BrF3′H* | Fw | CTCGCCGGAGTATTCAACAT | 106 | Bra009312 |  |
|  | Rv | AAGCGTCGAACCTCTTGTGT |  |  |  |
| *BrFLS1* | Fw | GTACCCGCGCATACTGATCT | 199 | Bra009358 |  |
|  | Rv | CCTTATCCACCGTCGTCCTA |  |  |  |
| *BrDFR* | Fw | AGGCAAAGACCGTACGAAGA | 186 | Bra027457 |  |
|  | Rv | CGTAATCCCAAGCTGCTTTC |  |  |  |
| *BrANS* | Fw | AAAAAGCGGAATCAGCTCAA | 166 | Bra013652 |  |
|  | Rv | GCATTTCTCACGGATGGTTT |  |  |  |
| *BrUGT75C1* | Fw | CCTCTCACAGCCACCTCTTC | 177 | Bra038445 |  |
|  | Rv | TCACCAAGACTTTGGGGTTC |  |  |  |
| *BrUGT78D2* | Fw | AAAGTGCCGTTTGTTTGGTC | 155 | Bra023594 |  |
|  | Rv | CAATGCGTCACAAAAACACC |  |  |  |
| *BrMYB12* | Fw | ACGTGTTGACATCGTGTGGT | 174 | Bra000453 |  |
|  | Rv | CCGTGAACGCATTATTGTTG |  |  |  |
| *BrMYB111* | Fw | TCCAAACCAATGGTGAAGGT | 194 | Bra037419 |  |
|  | Rv | TGCAATATGCGACCATCTGT |  |  |  |
| *BrPAP2* | Fw | CTCCATGCCTTGGACTCAAC | 150 | Bra001917 |  |
|  | Rv | CAGGCTGTTTGCTCTCCTCT |  |  |  |
| *BrMYB113* | Fw | TCTATGCCTTGGACACAACG | 150 | Bra004162 |  |
|  | Rv | GCTGGATCTTGGCTCTCATC |  |  |  |
| *BrTT8* | Fw | AGCGACGCAGAAGAGAGAAG | 173 | Bra037887 |  |
|  | Rv | CTTTTGGTTTGGCTCGTGAT |  |  |  |
| *BrTTG1* | Fw | CGTCCTCAACAACAGCAAGA | 181 | Bra009770 |  |
|  | Rv | GATGTCGTGGACCTCCTTGT |  |  |  |
| *BrTT19* | Fw | GGCAGCTTGTCCTCAAAGAG | 197 | Bra008570 |  |
|  | Rv | TCGCGTAGTATCTCGCAATG |  |  |  |
| *BrActin-1* | Fw | AATGGTGAAGGCTGGTTTTG | 177 | JN120480.1 | [60] |
|  | Rv | CCATGCTCGATCGGATACTT |  |  |  |
| *BrActin-2* | Fw | AATGGTACCGGAATGGTCAA | 119 | FJ969844.1 | NCBI |
|  | Rv | TCCTTCTGGTTCATCCCAAC |  |  |  |
| *BrActin-3* | Fw | TATCCGATCGAGCACGGTAT | 101 | EU012495.1 | NCBI |
|  | Rv | GGGTGCTCTTCAGGTGCTAC |  |  |  |
| COS1078 | Fw | ATGCCATTCTAAACACAAACCAC |  | Bra029903 | [73] |
|  | Rv | AAGGGAACATTGTGACGTAAAGC |  |  |  |

***Table S3b. Oligonucleotide primers used for qRT–PCR analysis of*** B. oleracea

| **Gene name** |  | **Primer sequences (5'----------------3')** | **Product size (bp)** | **Gene ID/ Acc. no.** | **References** |
| --- | --- | --- | --- | --- | --- |
| *BolPAL1* | Fw | GCAGAGCAACACAACCAAGA | 187 | Bol025522 |  |
|  | Rv | TGGCCACTTGAGAGACAGTG |  |  |  |
| *BolPAL2* | Fw | CGAGGAAGCCTTCAAACAAG | 150 | Bol005411 |  |
|  | Rv | TCCGCTAAAACCGATTGAAC |  |  |  |
| *BolC4H* | Fw | GGTTCTCGCCACAATGATCT | 184 | Bol004608 |  |
|  | Rv | TAGGTCTCGTTGACCCATCC |  |  |  |
| *Bol4CL1* | Fw | TGTGAAATCTGGTGCTGCTC | 181 | Bol031583 |  |
|  | Rv | AACCACCGTACCACAAGCTC |  |  |  |
| *BolCHS* | Fw | GTGGTGGTCGAAGTCCCTAA | 199 | Bol043396 |  |
|  | Rv | GAAGACCGAGGAGCTTTGTG |  |  |  |
| *BolCHI* | Fw | CACGGGTTCGTTTGAGAAGT | 200 | Bol008652 |  |
|  | Rv | AGAGAATCGAAGCACCAGGA |  |  |  |
| *BolF3H* | Fw | TTCTTCGCCTTACCTCCTGA | 198 | Bol010585 |  |
|  | Rv | CGTCACTTTCACCCATCCTT |  |  |  |
| *BolF3′H* | Fw | CTCTCGCCGGAGTATTCAAC | 173 | Bol043829 |  |
|  | Rv | GTCCGTGTGCTTTTGATCCT |  |  |  |
| *BolFLS1* | Fw | TCGAAGGGTATGGAACGAAG | 167 | Bol043773 |  |
|  | Rv | CTTCACATGCAACGCGTACT |  |  |  |
| *BolDFR* | Fw | CCGGCGCATCAGGATTCATT | 210 | Bol035269 |  |
|  | Rv | CGCCGTCGCATCCGTTTAT |  |  |  |
| *BolANS* | Fw | GCCCAAGACACCAAGTGATT | 194 | Bol042059 |  |
|  | Rv | GCTGAGGACATTTCGGGTAA |  |  |  |
| *BolUGT75C1* | Fw | CCTCTCACAGCCACCTCTTC | 177 | Bol027055 |  |
|  | Rv | TCACCAAGACTTTGGGGTTC |  |  |  |
| *BolUGT78D2* | Fw | TACAGTCATGACGCCGTCTC | 196 | Bol021317 |  |
|  | Rv | CGTTGCTTCGTGTTTTAGCA |  |  |  |
| *BolMYB12* | Fw | ATTCAGTCCAACGGTGAAGG | 184 | Bol029626 |  |
|  | Rv | ACCACCTGTTTCCCAAAGTG |  |  |  |
| *BolMYB111* | Fw | GGCGGCTCATGAGTAGTTTC | 104 | Bol032351 |  |
|  | Rv | GTCCCCACTCTCACACACCT |  |  |  |
| *BolPAP2* | Fw | AGCAAAGAGGCTCAGATGGA | 151 | Bol028756 |  |
|  | Rv | TCGACGTGTGGTTTTCTTCA |  |  |  |
| *BolMYB113* | Fw | TGCCAGAAGCTGACATTGTT | 150 | Bol012528 |  |
|  | Rv | GCAAACTTTCCCACCACCTA |  |  |  |
| *BolTT8* | Fw | CCAATAGTTTAGATACACACATGGACATG | 159 | BH450920 | [11] |
|  | Rv | TCTTTGACATTCTCAACTCTCCACGATAT |  |  |  |
| *BolTTG1* | Fw | CCTCAACAACAGCAAGACGA | 178 | Bol022420 |  |
|  | Rv | GATGTCGTGGACCTCCTTGT |  |  |  |
| *BolTT19* | Fw | CGACGTGGAGACCCATTACT | 188 | Bol021325 |  |
|  | Rv | TGAATTCATCACCAGCCAAA |  |  |  |
| *BolActin-1* | Fw | AATGGTCAAGGCTGGTTTTG | 108 | Bol022870 |  |
|  | Rv | TCCTTCTGGTTCATCCCAAC |  |  |  |
| *BolActin-2* | Fw | CTGTGACAATGGTACCGGAATG | 62 | AF044573 | [11] |
|  | Rv | ACAGCCCTGGGAGCATCA |  |  |  |
| *BolActin-3* | Fw | TTCCCTGGGATTGCTGATAG | 116 | Bol005539 |  |
|  | Rv | GACCCTCCAATCCAGACAGA |  |  |  |

11. Yuan Y, Chiu L-W, Li L: **Transcriptional regulation of anthocyanin biosynthesis in red cabbage**. *Planta* 2009, **230**(6):1141

60. Xie Q, Hu Z, Zhang Y, Tian S, Wang Z, Zhao Z, Yang Y, Chen G: **Accumulation and molecular regulation of anthocyanin in purple tumorous stem mustard (*Brassica juncea* var. tumida Tsen et Lee)**. *Journal of Aagricultural and Food Chemistry* 2014, **62**(31):7813-7821.

73. Jeong Y-M, Chung W-H, Chung H, Kim N, Park B-S, Lim K-B, Yu H-J, Mun J-H: **Comparative analysis of the radish genome based on a conserved ortholog set (COS) of Brassica**. *Theoretical and applied genetics* 2014, **127**(9):1975-1989
