# Supplementary figures and images for: Transcriptional regulation of anthocyanin biosynthesis in a high-anthocyanin resynthesized Brassica napus cultivar
Source: J Biol Res (Thessalon). 2018 Nov 26;25:19. doi: 10.1186/s40709-018-0090-6 (PMC6258291; doi:10.1186/s40709-018-0090-6)

## Slide 1
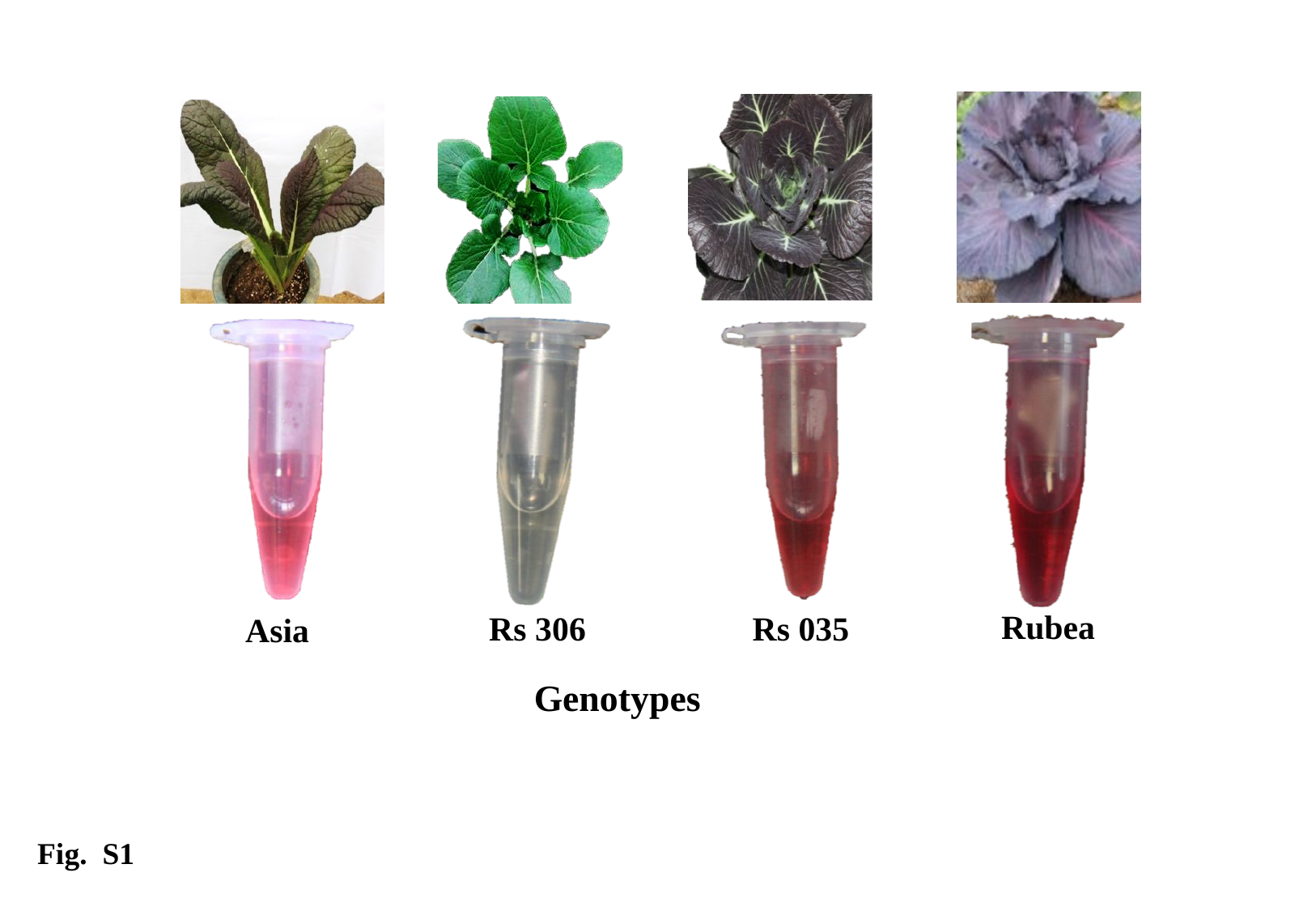

Rubea
Rs 035
Rs 306
Asia
Genotypes
Fig. S1

## Slide 2
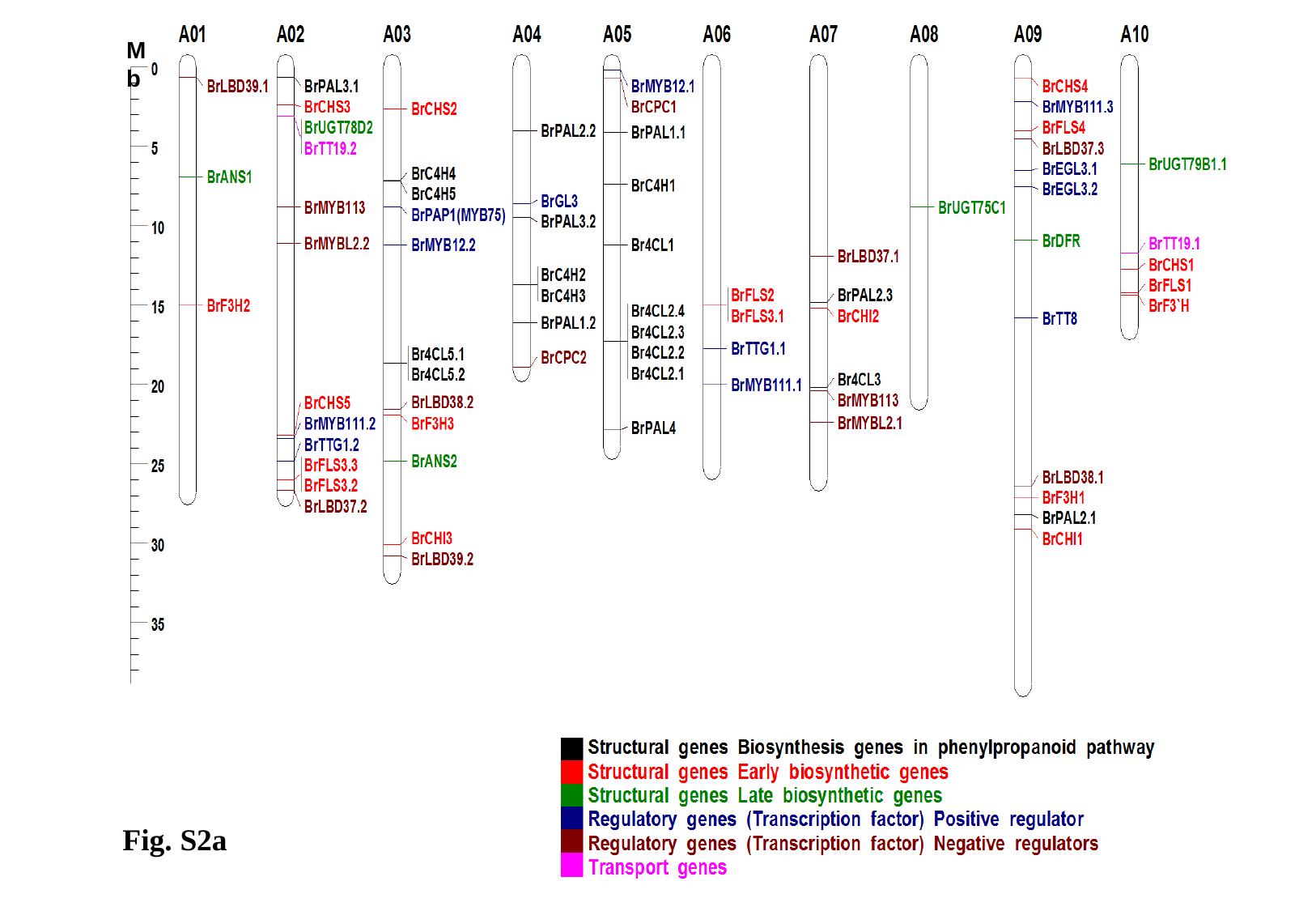

Mb
Fig. S2a

## Slide 3
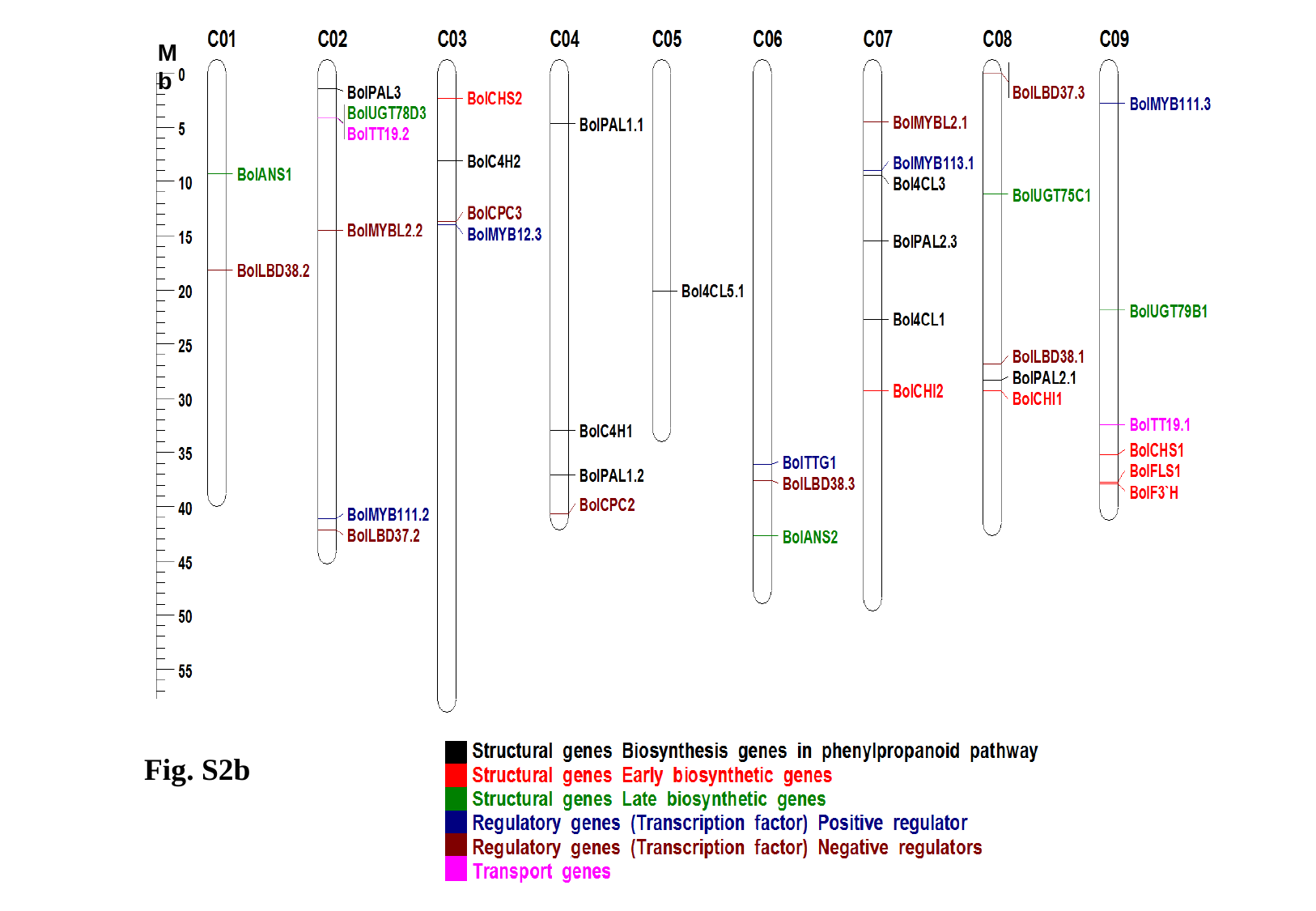

Mb
Fig. S2b

## Slide 4
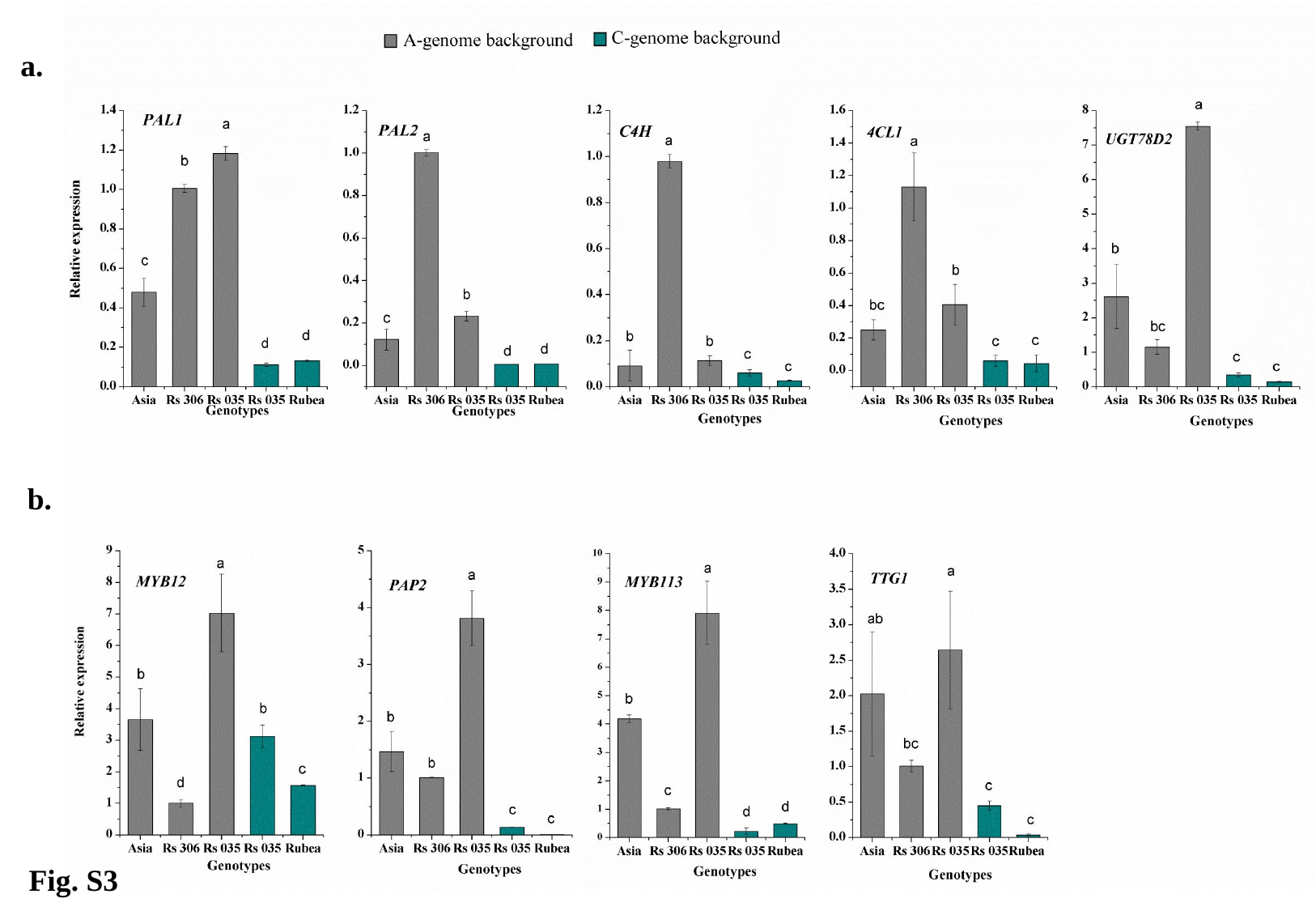

a.
b.
Fig. S3

Supplement: Supplementary file 5 — Additional file 5: Figure S1. Morphological distinctiveness and anthocyanin contents of four Brassica lines. The visual phenotype (a) and 4th-leaf total anthocyanin contents of 6-week-old plants (b) of the parental lines Asia (B. rapa) and Rubea (B. oleracea), the red allopolyploid resynthesized B. napus (Rs035), and the control green allopolyploid resynthesized B. napus (Rs306) are shown. Figure S2. Chromosomal distributions of the putative anthocyanin biosynthesis genes in (a) Brassica rapa and (b) Brassica oleracea. The chromosome number is indicated at the top of each chromosome. The scale (left) is in megabases (Mb). The colors represent the putative structural genes of the phenylpropanoid pathway (black), early-biosynthesis (red), late-biosynthesis (green), positive-regulatory (blue), and negative-regulatory (brown) genes. Genes assigned to scaffold sequences are not shown on the physical maps. Figure S3. Relative gene expression of anthocyanin pathway genes which did not show striking expression changed compared among the studied four lines, (a) structural and (b) regulatory and transporter genes. [file 40709_2018_90_MOESM5_ESM.ppt]
